# Transition metal-catalyzed cross-coupling reactions of *N*-aryl-2-aminopyridines

**DOI:** 10.1039/d4ra08384a

**Published:** 2025-01-14

**Authors:** Fatemeh Doraghi, Lina Rezainia, Mohammad Hossein Morshedsolouk, Hamed Navid, Bagher Larijani, Mohammad Mahdavi

**Affiliations:** a Endocrinology and Metabolism Research Center, Endocrinology and Metabolism Clinical Sciences Institute, Tehran University of Medical Sciences Tehran Iran momahdavi@tums.ac.ir; b School of Chemistry, College of Science, University of Tehran Tehran Iran

## Abstract

Due to the presence of the pyridyl directing group, *N*-aryl-2-aminopyridines can quickly form stable complexes with metals, leading to cyclization and functionalization reactions. A large number of N-heterocycles and nitrogen-based molecules can be easily constructed *via* this direct and atom-economical cross-coupling strategy. In this review, we have highlighted the transformations of *N*-aryl-2-aminopyridines in the presence of various transition metal catalysts, such as palladium, rhodium, iridium, ruthenium, cobalt and copper.

## Abstract 2

Owing to the pyridyl directing group, *N*-aryl-2-amnopyridines form stable metal complexes towards N-heterocycles and nitrogen-based compounds. We reviewed transition metal-catalyzed annulation and functionalization reactions of this synthon.

## Graphical abstract

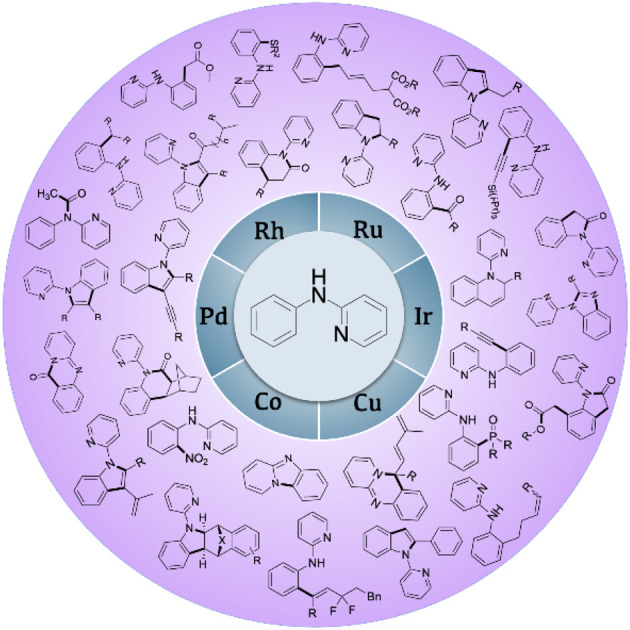

## 1. Introduction

Transition metal-catalyzed directing group-assisted heteroannulations of amines with other coupling partners have emerged as a powerful and efficient tool.^1–5^ N-Heterocyclic compounds are the main nuclei in functional materials,^6,7^ pharmaceuticals^8–11^ and natural products.^12–14^ Consequently, various straightforward and affordable routes have been developed for synthesizing bioactive N-heterocycles. Among them, nitrogen-directing group-assisted substrates, leading to *ortho*-selective C–H activation are very popular. The nitrogen auxiliary groups form a variety of stable metal complexes and are then easily removed or undergo further transformations.

**Scheme 1:**
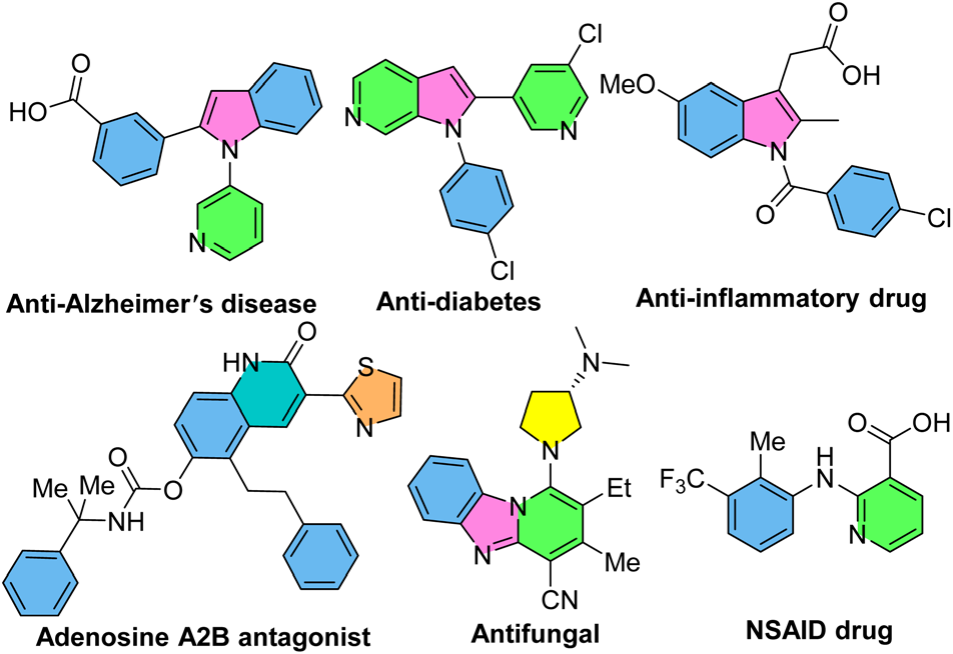
Some pharmaceutically important nitrogen-containing compounds.

*N*-Aryl-2-aminopyridines, which are readily prepared by the coupling of anilines with 2-bromopyridines,^15–17^ are extensively used as substrates in the context of C–H activation. Owing to the presence of the pyridyl as a directing group, *N*-aryl-2-aminopyridines can easily incorporate into the chelation-assisted C–H bond functionalization. Various transition metals, such as palladium, rhodium, iridium, ruthenium, cobalt and copper can be involved in the formation of five-, six- and seven-membered complex intermediates with the nitrogen-pyridyl group through the coordination and C–H bond activation process. A diverse range of bioactive N-heterocycles, including indoles, carbazoles, quinolinones, benzimidazoles, indolinones, imidazopyridines and functionalized *N*-aryl-2-aminopyridines can be obtained *via* annulation or functionalization of *N*-aryl-2-aminopyridines.^18–23^ Some of biologically active compounds containing these valuable cores are depicted in Scheme 1.

Considering the importance of *N*-aryl-2-aminopyridine derivatives, we have highlighted the developments related to these synthons. In this context, we have discussed the annulation and functionalization of *N*-aryl-2-aminopyridine in the presence of transition metal catalysts. We have categorized this review based on the metal type to provide a better insight into the behaviour of each metal catalyst in the presence of *N*-aryl-2-aminopyridines.

## 2. Transition metal-catalyzed cross-coupling of *N*-aryl-2-aminopyridines

### 2.1. Pd-catalyzed cross-coupling of *N*-aryl-2-aminopyridines

#### 2.1.1. Annulation

**Scheme 2:**
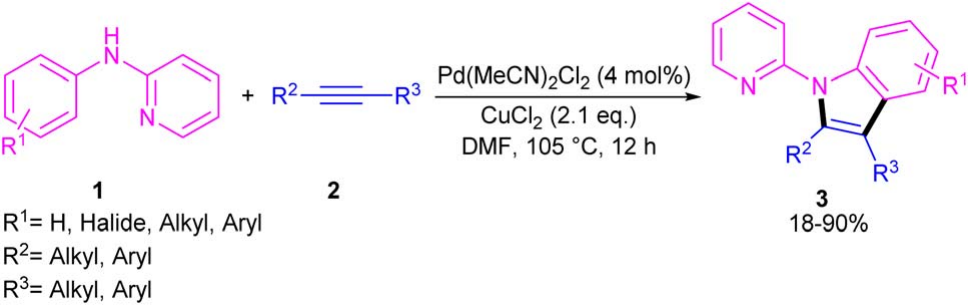
Pd(ii)-catalyzed annulation of *N*-aryl-2-aminopyridine and alkynes.

**Scheme 3:**
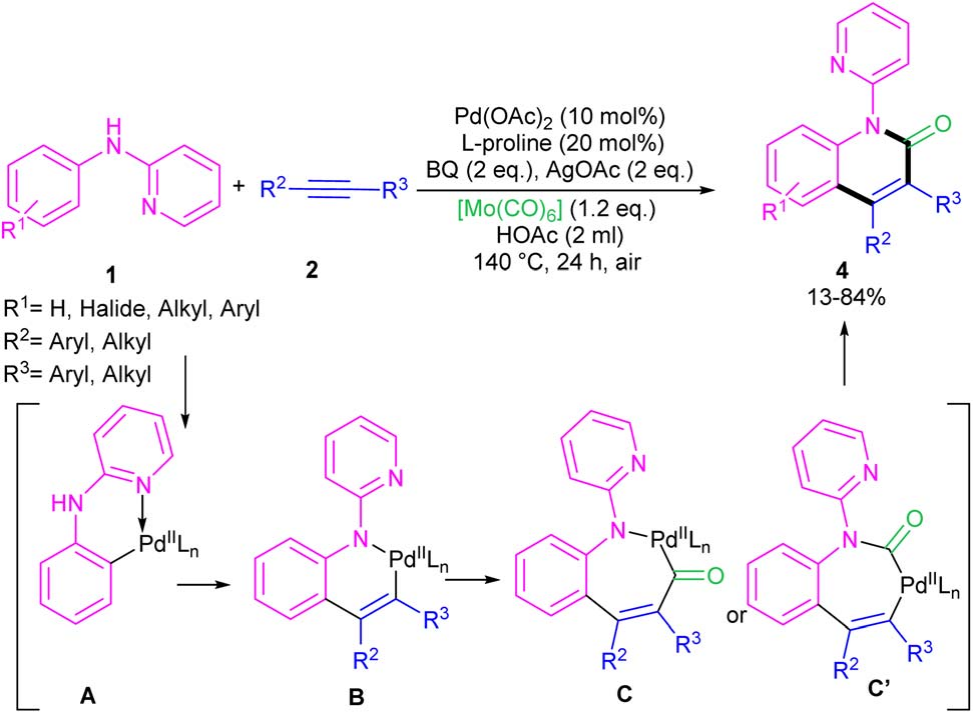
Pd(ii)-catalyzed annulation of *N*-aryl-2-aminopyridine, alkynes and Mo(CO)_6_.

In 2011, Li's research group performed a study on the Pd(ii)-catalyzed synthesis of *N*-(2-pyridyl)indole frameworks 3 through the annulation of *N*-aryl-2-aminopyridines 1 with internal alkynes 2 (Scheme 2).^24^ In this synthetic method, 4 mol% of Pd(MeCN)_2_Cl_2_ as a catalyst, 1.2 equiv. of CuCl_2_ and O_2_ atmosphere as the oxidants were used in DMF solvent. It should be noted that this palladium(ii) system did not prove effective for the reaction of *N*-aryl-2-aminopyridines with acrylates towards *N*-(2-pyridyl)quinolones, as these products were achieved in the presence of a Rh(iii) catalyst. However, in Wu's laboratory, *N*-(2-pyridyl)quinolones were well synthesized from the annulation of *N*-aryl-2-aminopyridines 1, internal alkynes 2 and Mo(CO)_6_ as a CO source through a (3 + 2 + 1)-cycloaddition process (Scheme 3).^25^ The currently accepted mechanism involved the initial C–H activation of substrate 1 with Pd(ii), followed by the insertion of an alkyne to produce intermediate B. Subsequent coordination and insertion of CO to B led to intermediate C or C′, which upon reductive elimination gave product 4. The generated Pd(0) was re-oxidized by BQ and AgOAc to the active Pd(ii) species.

**Scheme 4:**
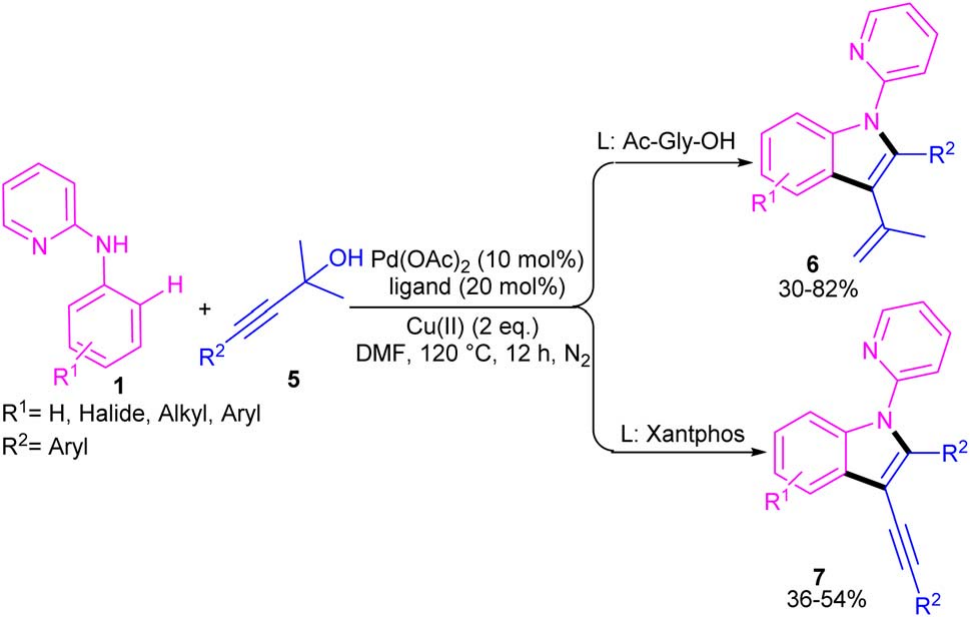
Pd(ii)-catalyzed annulation of *N*-aryl-2-aminopyridines with propargylic alcohols.

**Scheme 5:**
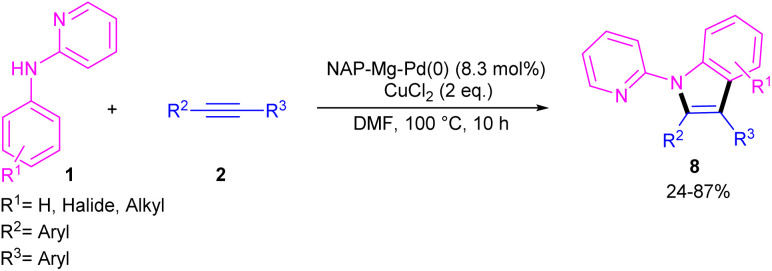
NAP–Mg–Pd(0) catalyzed oxidative coupling of *N*-aryl-2-aminopyridines with alkynes.

In 2021, Zhou's group reported the assembly of *N*-pyridoindoles 6, 7 by using *N*-aryl-2-aminopyridines 1 and propargylic alcohols 5 (Scheme 4).^26^ The catalytic system involved Pd(OAc)_2_ as a catalyst, Cu(OAc)_2_ or CuSO_4_ as an oxidant, Ac-Gly-OH or xantphos as a ligand. Other metal catalysts, such as Co(OAc)_2_·4H_2_O, Ni(OAc)_2_·4H_2_O, [RuCl_2_(*p*-cymene)]_2_ and [Cp*RhCl_2_]_2_ were not workable as much as Pd(OAc)_2_. The indole products were isolated in moderate to high yields (30–82%). Another Pd-catalyzed cyclization reaction between *N*-aryl-2-aminopyridines and alkynes access to indoles was reported by Hu and co-workers.^27^ PdCl_2_ as a catalyst, Cu(TFA)_2_·*x*H_2_O as an oxidant, and DTBP as a co-oxidant efficiently catalyze this (3 + 2)-cycloaddition process. Nano magnesium oxide can stabilize Pd(0) leading to a heterogeneous nanocatalyst for the C–H activation/annulation of *N*-aryl-2-aminopyridines 1 with alkynes 2 (Scheme 5).^28^ As an oxidant, CuCl_2_ was more practical than the Ag salts, TBHP or O_2_ atm. Many *N*-(2-pyridyl)indole products 8 were isolated in good yields by simple operation. Besides, *N*-aryl-2-aminoquinoline and *N*-aryl-2-aminopyrimidine were also yielded the products in 24% and 77%, of yields, respectively. The nanocatalyst was then investigated for its recyclability, which efficiently catalyze the reaction for four cycles without significant loss of catalytic activity. So, it can be concluded that this nanocatalyst acts better than the palladium homogeneous catalysts.

**Scheme 6:**
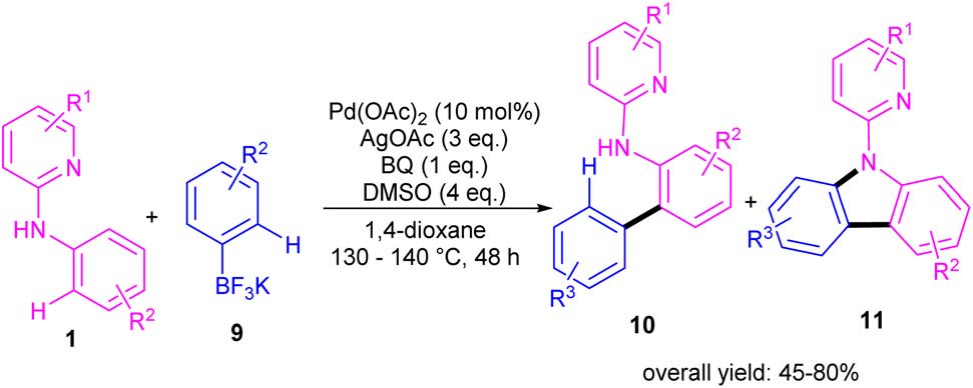
Pd(ii)-catalyzed cross-coupling of *N*-phenylpyridin-2-amines with potassium aryltrifluoroborates.

During an investigation of carbazole synthesis by means of a Pd(ii)-catalyzed, one-pot reaction, Wu's group applied a procedure to *N*-aryl-2-aminopyridines 1 to achieve 9-(pyridin-2-yl)-9*H*-carbazoles 11 in moderate to high yields (Scheme 6).^29^ The reaction involved *N*-aryl-2-aminopyridines and potassium aryltrifluoroborates in the presence of a Pd(OAc)_2_ catalyst, AgOAc oxidant and benzoquinone (BQ) ligand. Using starting materials with different functional groups resulted in different ratios of products 10 and 11, indicating the low selectivity of this reaction. The BQ ligand in collaboration with Ag(i) had an important role in not only the transmetalation but also reductive elimination.

**Scheme 7:**
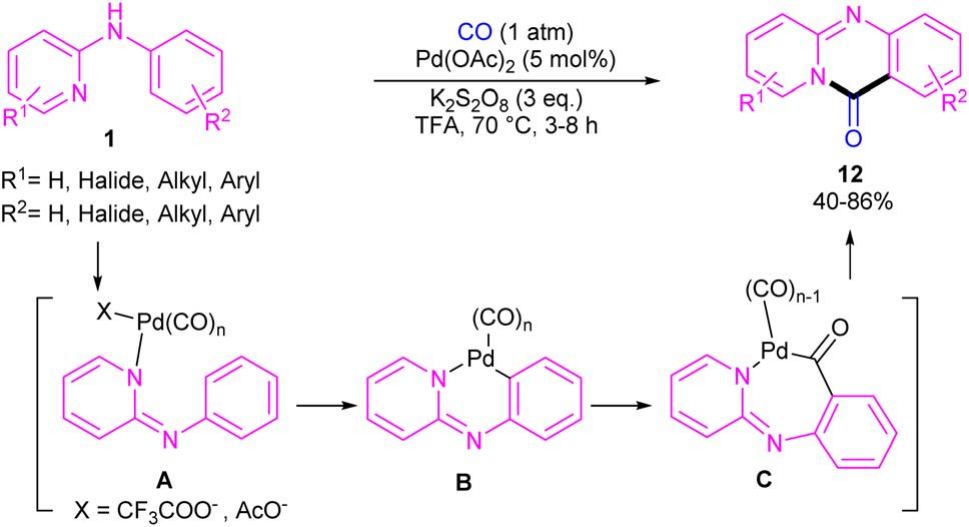
Pd(ii)-catalyzed C(sp^2^)–H pyridocarbonylation of *N*-aryl-2-aminopyridines.

**Scheme 8:**
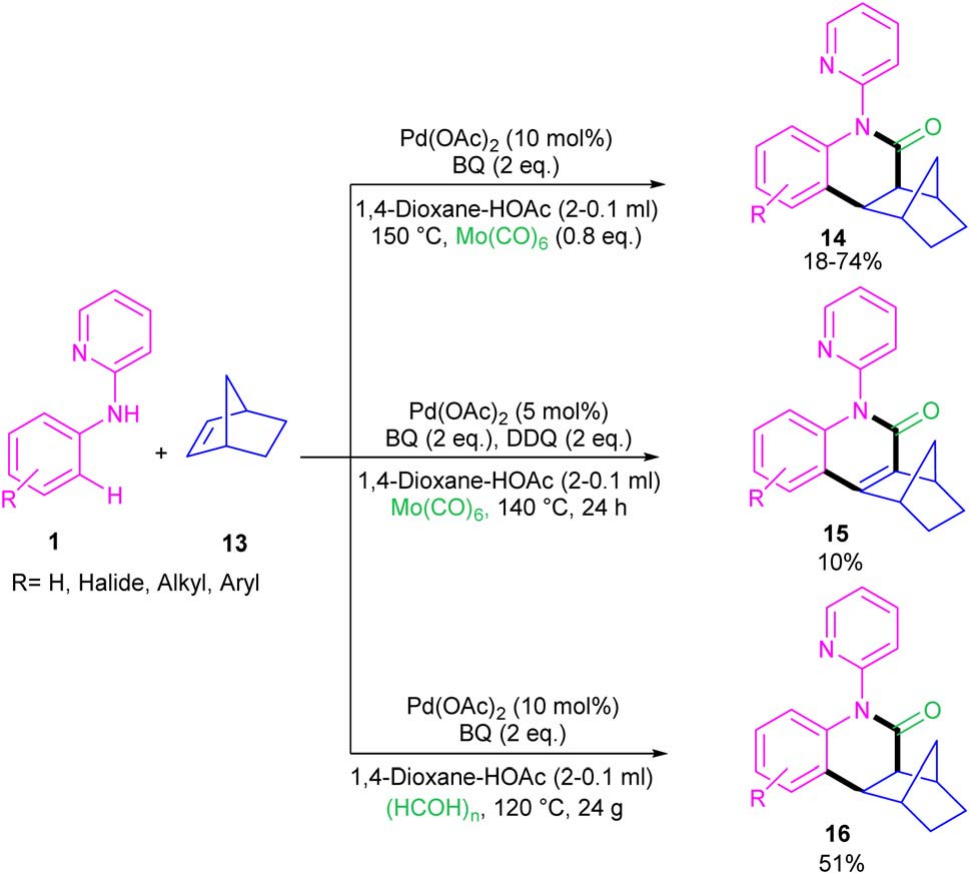
Pd(ii)-catalyzed carbonylative C–H activation of *N*-aryl-2-pyridinamines with norbornene.

For preparing 11*H*-pyrido[2,1-*b*]quinazolin-11-ones 12, Zhu *et al.* used Pd(OAc)_2_ as a catalyst and CO gaseous to perform carbonylative intramolecular cyclization of *N*-aryl-2-aminopyridines 1 (Scheme 7).^30^ The pyridyl nitrogen acted not only as a directing group for the metal but also as an intramolecular nucleophile. The reaction was found to proceed through intermediate A, B and C. The Pd(ii) complex A was generated from the chelation of the nitrogen with the CO ligated Pd(ii), which upon electrophilic cyclopalladation on the phenyl ring gave intermediate B*via* the C–H bond cleavage. After that, palladacycle C was formed *via* migratory insertion of a coordinated CO into the aryl–Pd bond, which after reductive elimination liberated the final product 12. Later, Wu and co-workers reported the synthesis of 5-(pyridin-2-yl)-hexahydro-7,10-methanophenanthridin-6(5*H*)-ones from *N*-phenylpyridin-2-amine 1 and norbornene 13 as substrates using Pd(OAc)_2_ as a catalyst and Mo(CO)_6_ as a solid CO source (Scheme 8).^31^ Overall catalytic cycle involved Pd(ii) insertion into the aromatic C–H bond, alkene coordination/insertion, CO coordination/insertion, and final reductive elimination. The carbonylation/cyclization reaction was also carried out by changing the BQ to DDQ as an oxidant or using paraformaldehyde as a CO source. However, DDQ can lead to the over oxidized product 15.

**Scheme 9:**
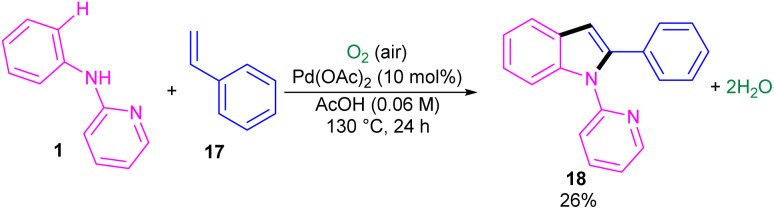
Pd(ii)-catalyzed cyclization of anilines with vinyl azides.

**Scheme 10:**
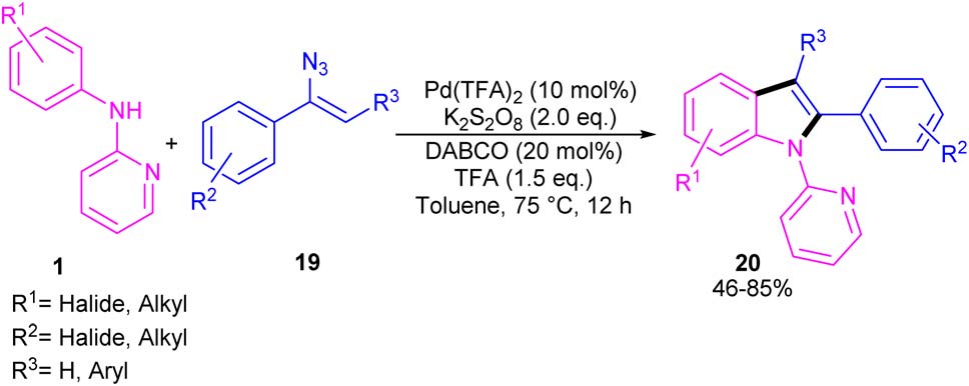
Pd(ii)-catalyzed cyclization of anilines with vinyl azides.

**Scheme 11:**
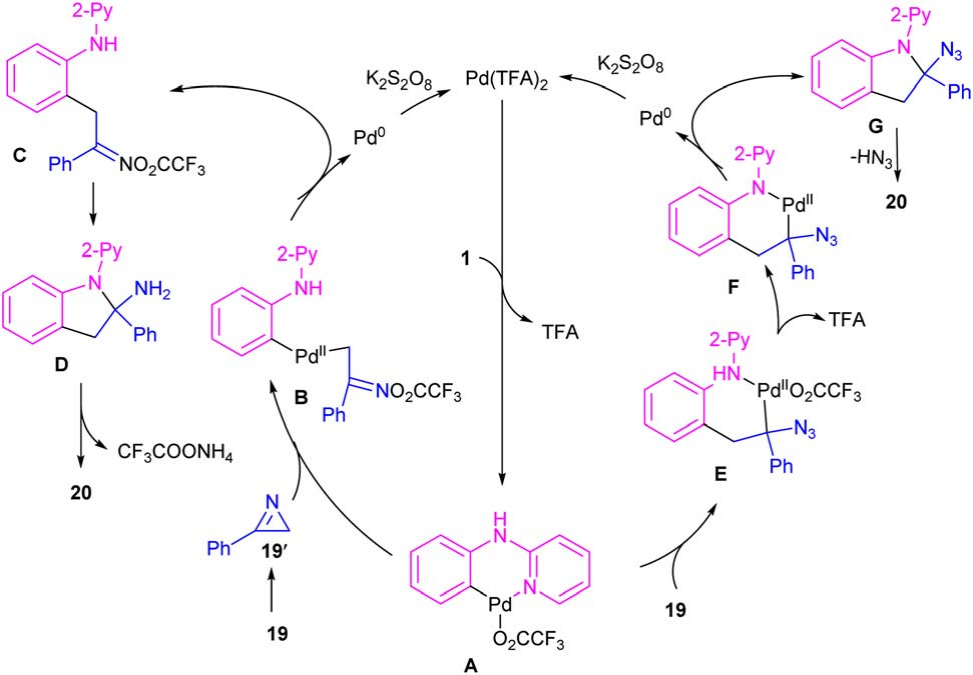
Plausible mechanism for Pd(ii)-catalyzed cyclization of anilines with vinyl azides.

The first report on the incorporation of olefins in intramolecular cyclization without the need of any additional directing group was performed by Maiti and his research team in 2014 (Scheme 9).^32^*N*-Phenyl-2-aminopyridine 1 showed moderate reactivity in this reaction, resulting in 26% of yield of product 18. In 2018, Wang, Cui and co-workers designed a strategy to achieve 2-arylindoles 20*via* palladium-catalyzed reaction of *N*-phenyl-2-aminopyridine 1 with (1-azidovinyl)-benzene 19 (Scheme 10).^33^ The mechanism started with a concerted metalation/deprotonation (CMD) process to access the palladacycle A. This intermediate could be moved through two pathways. In path I, the six-membered intermediate A underwent migratory insertion with vinyl azide 19, and a subsequent elimination of TFA to yield another six-membered palladacycle F. Oxidative cyclization towards the indoline G, followed by the elimination of HN_3_ furnished indole 20. Meanwhile, K_2_S_2_O_8_ oxidized Pd(0) to the active Pd(ii) species to restart the next catalytic cycle. In path II, vinyl azide 19 transformed to 2*H*-azirine 19′ by the help of DABCO. Then, migratory insertion into palladacycle A, and subsequent reductive elimination generated intermediate C. The generated Pd(0) species could be reoxidized to Pd(ii) species. At last, C was subjected to an intramolecular nucleophilic addition to form intermediate D, which by further deamination delivered indole 20 (Scheme 11). It should be noted that intermediates C and D were determined by HRMS analysis, confirming the second pathway. In addition, KIE study (*K*_H_/*K*_D_ = 1.4) indicated that the C–H bond cleavage is involved in the rate-determining step.

**Scheme 12:**
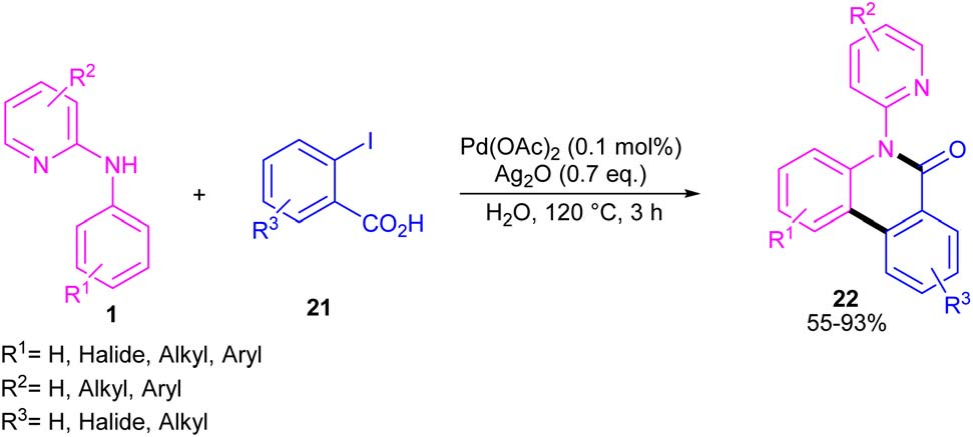
Pd(ii)-catalyzed cyclization of *N*-aryl-2-aminopyridines with 2-iodobenzoic acids.

**Scheme 13:**
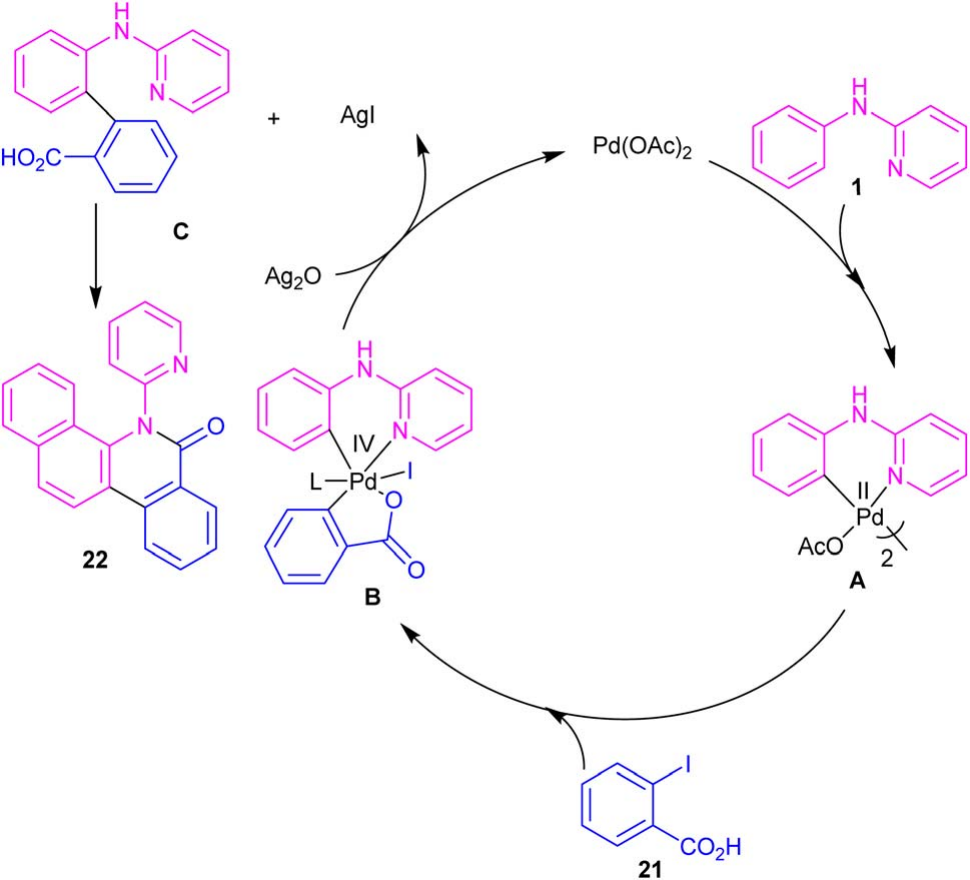
Catalytic cycle for Pd(ii)-catalyzed cyclization of *N*-aryl-2-aminopyridines with 2-iodobenzoic acids.

*N*-Aryl-2-aminopyridines 1 and 2-iodobenzoic acids 21 can react together in the presence of 0.1 mol% of Pd(OAc)_2_ and 0.7 equiv. of Ag_2_O in H_2_O as a green solvent to construct phenanthridinone scaffolds 22 (Scheme 12).^34^ The use of methyl 2-iodobenzoate and iodobenzene instead of 2-iodobenzoic acids did not result in any product, indicating the crucial role of the acid unit in the cyclization process. The isolation of the palladium intermediate I was successful, which in the reaction with 2-iodobenzoic acid led to the desired product in 91% yield. So, the reaction was found to move through the coordination of Pd(ii) to the N-atom of *N*-aryl-2-aminopyridine, followed by C–H activation to form a six-membered palladacycle dimer A. In the next step, carboxylate-assisted oxidative addition to the metal center to render a Pd(iv) intermediate B. Then, reductive elimination of B in the presence of Ag(i) furnished the *ortho*-arylated product C. Further intramolecular acylation gave phenanthridinone 22 (Scheme 13).

#### 2.1.2. Functionalization

##### 2.1.2.1. Alkynylation

**Scheme 14:**
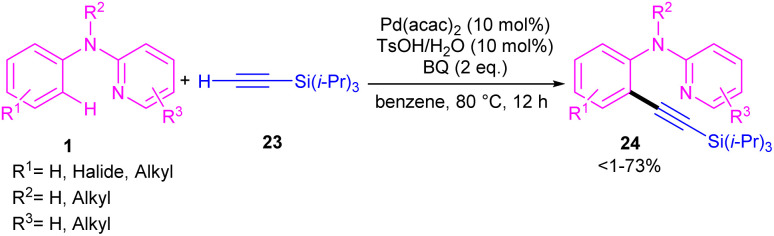
Pd(ii)-catalyzed reaction of *N*-aryl-2-aminopyridines with (triisopropylsilyl)acetylene.

**Scheme 15:**
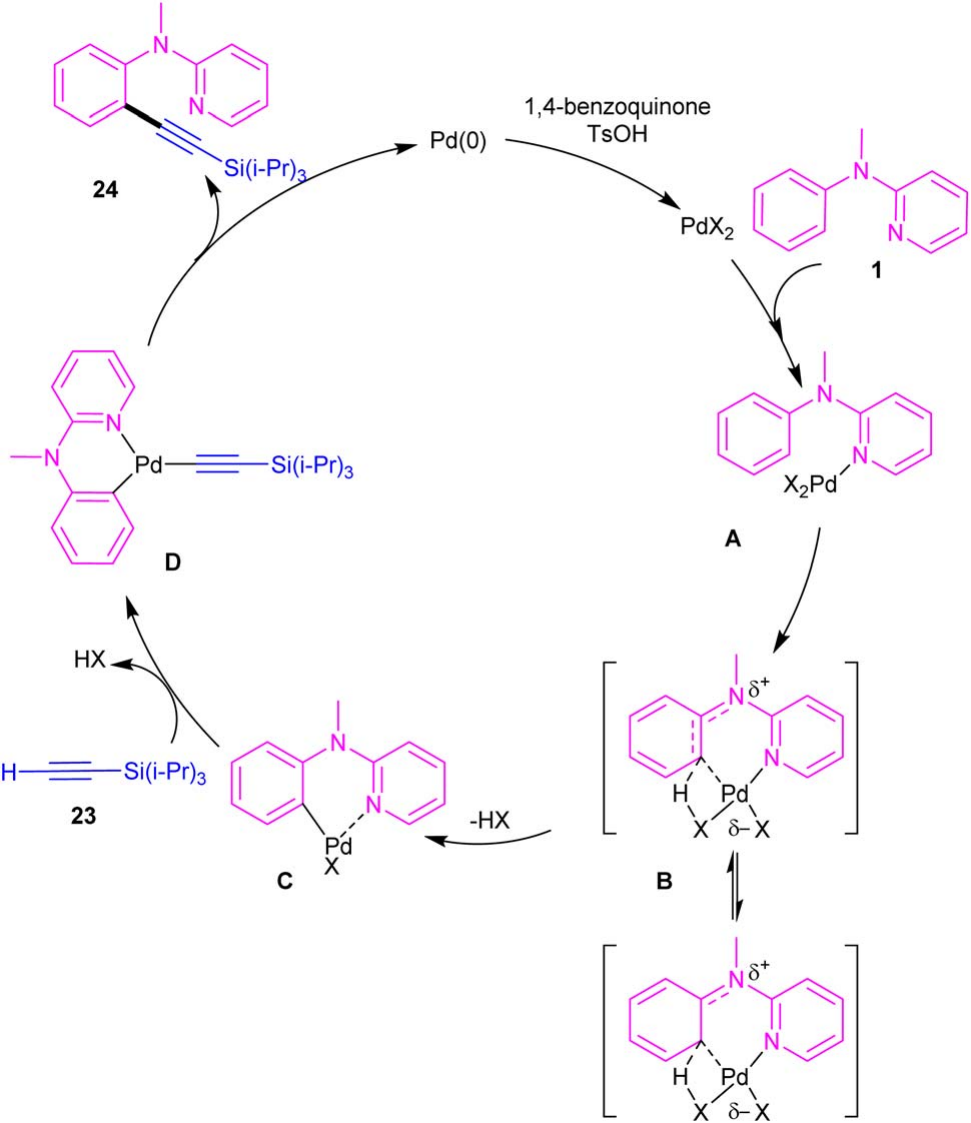
Proposed mechanism for Pd(ii)-catalyzed reaction of *N*-aryl-2-aminopyridines with (triisopropylsilyl)acetylene.

Chang and co-workers investigated the reaction of *N*-aryl-2-aminopyridines 1 with (triisopropylsilyl)acetylene 23 in the presence of the palladium catalysts (Scheme 14).^35^ Several palladium salts were explored [Pd(OAc)_2_, Pd(acac)_2_]; Pd(acac)_2_ proved to be the most effective, in 100 mol% at 80 °C and in benzene as solvent. The KIE experiments indicated that C(sp^2^)–H bond cleavage is related to the rate-determining step. To determine the role of the nitrogen of *N*-aryl-2-aminopyridine in the catalytic pathway, the authors replaced *N*-aryl-2-aminopyridine with *N*-methyldiphenylamine and 2-benzylpyridine as coupling reactants, resulting in no product. So, the reaction was proposed to carry out through intermediate A, followed by electrophilic metalation pathway to generate the arylpalladium π-complex B. It was found that the nitrogen linker between phenyl and 2-pyridyl moieties could stabilized B*via* equilibrating with B′. The conversion of B to the six-membered palladacycle C, and subsequent ligand exchange by acetylene afforded the arylpalladium acetylide intermediate D, which under reductive elimination delivered the alkynylated product 24. The Pd(0) could be oxidized to Pd(ii) in the presence of BQ and TsOH (Scheme 15).

##### 2.1.2.2. Acylation

**Scheme 16:**
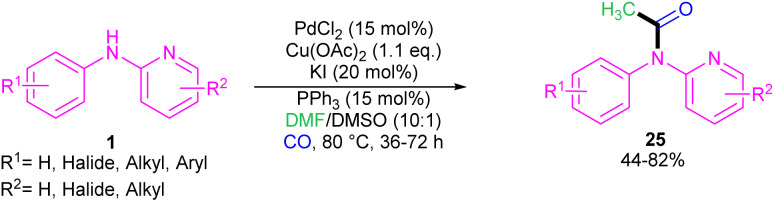
Pd(ii)-catalyzed acylation of *N*-phenyl-2-aminopyridines with DMF and CO.

**Scheme 17:**
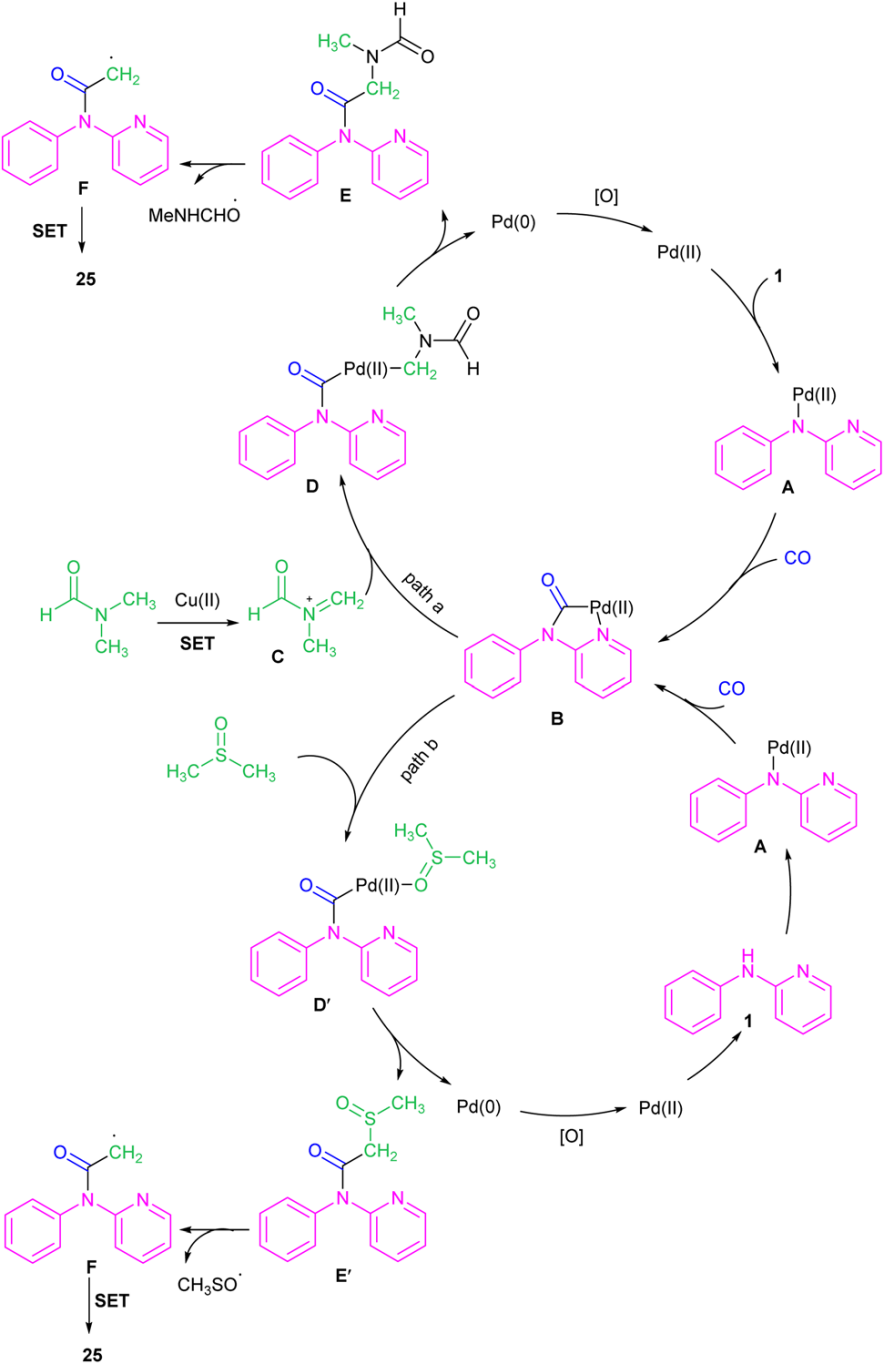
Rational mechanism for Pd(ii)-catalyzed acylation of *N*-phenyl-2-aminopyridines with DMF and CO.

In 2023, Jiang, Ji and co-workers realized that PdCl_2_ (15 mol%), Cu(OAc)_2_ (1.1 equiv.), KI (20 mol%), and PPh_3_ (15 mol%) with CO gaseous in DMF/DMSO at 80 °C constituted an efficient three-component system for the coupling of *N*-phenyl-2-aminopyridines with DMF and CO (Scheme 16).^36^ In this protocol, DMF served as a methyl source and CO as a carbonyl source. When DMSO was used as a sole solvent, it could act as a methyl source. Although mechanistic studies using DMSO-*d*_6_ showed that the methyl group in the product was originated from DMF not DMSO when the reaction performed in a mixture of both solvents, indicating DMF as a potential methyl source. The authors proposed a coupling mechanism in which Pd(0) inserted into the N–H bond of 1 to generate intermediate A, followed by the CO insertion to form intermediate B. In the meanwhile, DMF was oxidized to intermediate C, which then reacted with B to render intermediate D. The intermediate E was formed upon reductive elimination of D. Subsequent SET process in E afforded intermediate F, which was converted into product 25*via* another SET step. Additionally, a carbonylative acetylation can also be carried out using DMSO as a methyl source. Upon the coordination of DMSO to intermediate B, intermediate D′ was obtained, which underwent reductive elimination to yield intermediate E′. After that, the conversion of E′ to F*via* the C–S bond cleavage^37^ and subsequent SET process delivered product 25 (Scheme 17).

### 2.2. Rh-catalyzed cross-coupling of *N*-aryl-2-aminopyridines

#### 2.2.1. Annulation

**Scheme 18:**
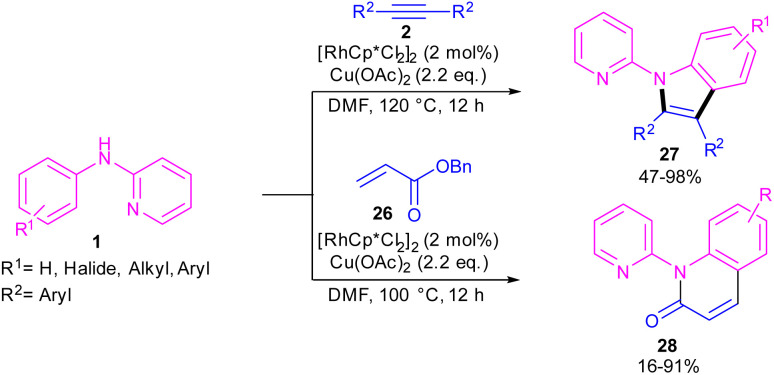
Rh(iii)-catalyzed C-annulation of *N*-aryl-2-aminopyridines with alkynes and acrylates.

In 2010, Li and co-workers reported an interesting method for producing *N*-(2-pyridyl)indoles 27 and *N*-(2-pyridyl)quinolones 28 through the annulation of two different reagents with *N*-aryl-2-aminopyridine 1 (Scheme 18).^38^ For this purpose, they treated *N*-aryl-2-aminopyridine 1 with alkynes 2 in the presence of 2 mol% of [RhCp*Cl_2_]_2_ to make *N*-(2-pyridyl)indoles 27*via* (3 + 2)-cycloaddition process. In another transformation, 4 mol% of [RhCp*Cl_2_]_2_ and acrylates 26 as coupling partners were used to perform (3 + 3)-cycloaddition with *N*-aryl-2-aminopyridine 1 towards the synthesis of *N*-(2-pyridyl)quinolones 28. In addition, under these catalytic conditions, the reaction of styrene with *N*-aryl-2-aminopyridine led to mono- and disubstituted olefination products.

**Scheme 19:**
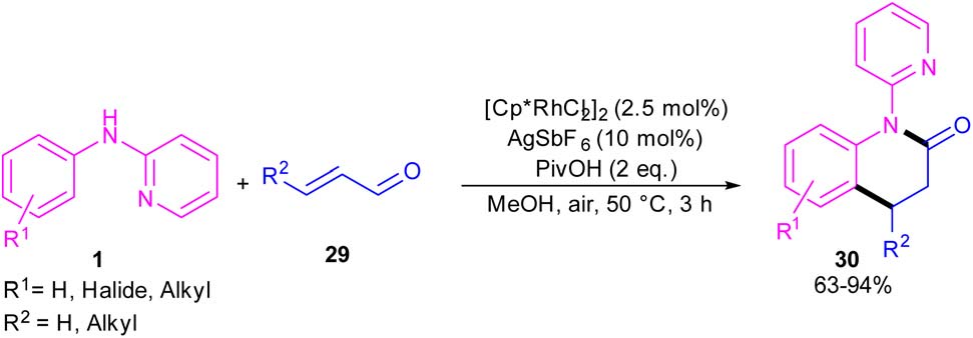
Rh(iii)-catalyzed C–H functionalization/cyclization reaction of *N*-arylpyridin-2-amines with α,β-unsaturated aldehydes.

**Scheme 20:**
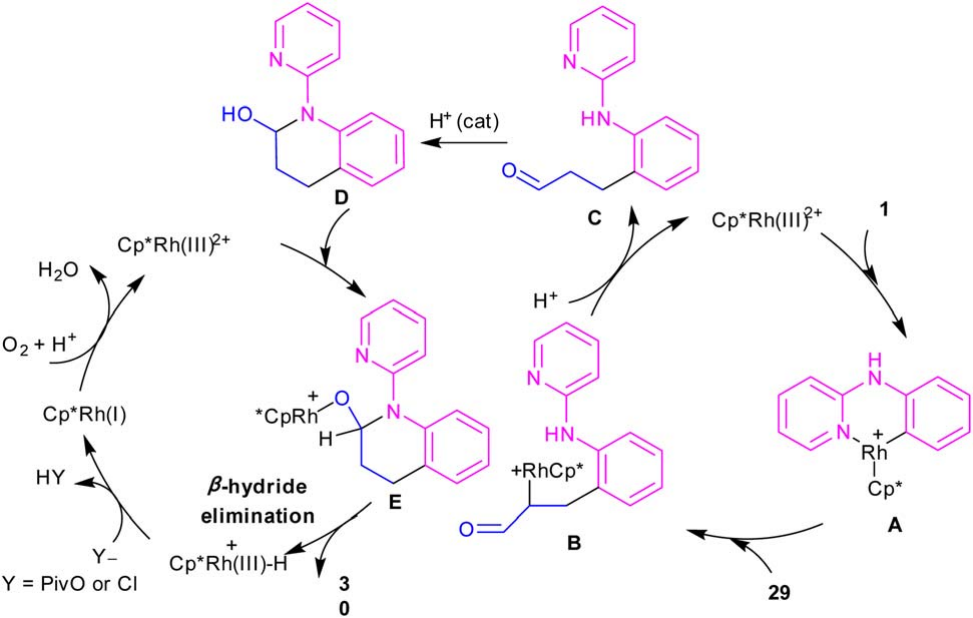
Rh(iii)-catalyzed C–H functionalization/cyclization reaction of *N*-arylpyridin-2-amines with α,β-unsaturated aldehydes.

In 2017, Huang and co-workers developed Rh(iii)-catalyzed C(sp^2^)–H functionalization/cyclization reaction of *N*-phenyl-2-aminopyridine and α,β-unsaturated aldehydes (Scheme 19).^39^ A series of dihydroquinolinones were well synthesized from both electron-rich and electron-poor *N*-aryl-2-aminopyridine substrates. According to the rhodium catalytic cycle in Scheme 20, pyridine-directed Rh(iii) insertion in the α-C(sp^2^)–H bond of *N*-aryl-2-aminopyridine 1 to produce a six-membered rhodacycle A. Then, propenal 29 inserted into A to form intermediate B, which was protonated to generate intermediate C. Acid-catalyzed intramolecular nucleophilic addition of the amine onto the carbonyl group to yield α-hydroxyl tetrahydroquinoline D. Afterward, through a ligand exchange between Cp*Rh(iii)^2+^ and D, a rhodium intermediate E was obtained, which underwent β-hydride elimination to afford the tetrahydroquinoline product 30 and Cp*Rh(iii)^+^–H. The interaction of the anion Y^−^ and Cp*Rh(iii)^+^–H resulted in the loss of a HY molecule, followed by reductive elimination to Cp*Rh and subsequent oxidation to the catalytically active Cp*Rh^2+^ species.

**Scheme 21:**
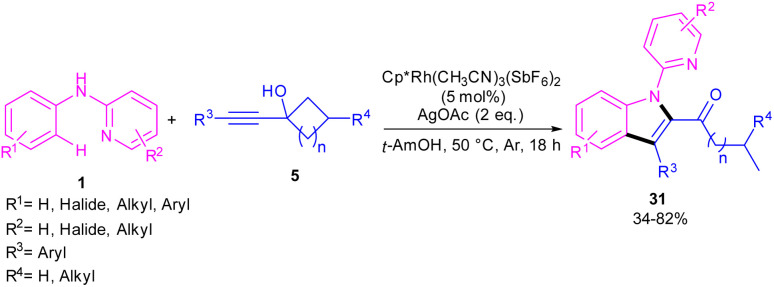
Rh(iii)-catalyzed cross-coupling/cyclization between *N*-aryl-2-pyridinamines and propargyl cycloalkanols.

**Scheme 22:**
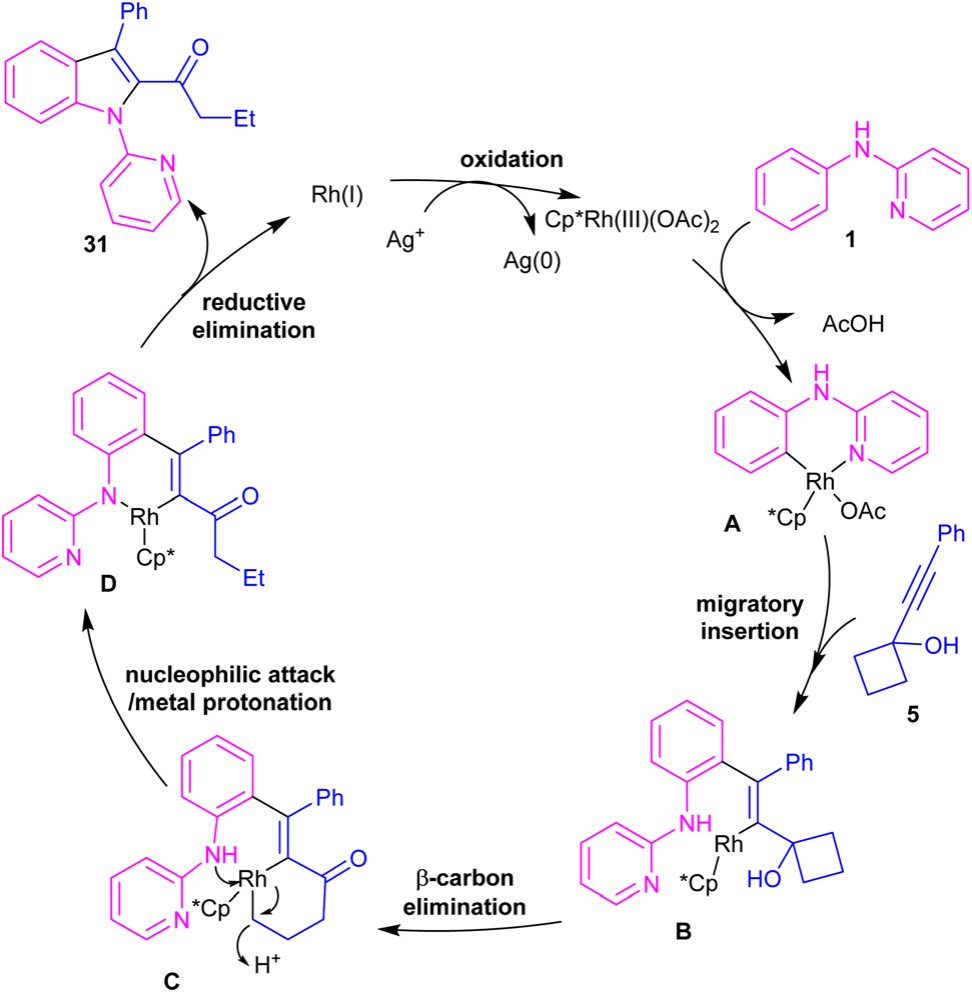
Possible mechanism of Rh(iii)-catalyzed cross-coupling/cyclization between *N*-aryl-2-pyridinamines and propargyl cycloalkanols.

In the same year, Zeng's research team developed a rhodium catalytic strategy for the cross-coupling/cyclization between *N*-aryl-2-pyridinamines 1 and propargylic cycloalkanols 5 (Scheme 21).^40^ A series of 1,2,3-trisubstituted indoles 31 were synthesized in moderate to high yields. Evaluating various transition metals, including Pd(OAc)_2_, Cp*Co(CO)I_2_, (*p*-cymeneRuCl)_2_, [Cp*IrCl_2_]_2_, RhCl_3_, [Cp*RhCl_2_]_2_ and Cp*Rh-(CH_3_CN)_3_(SbF_6_)_2_ showed that only rhodium complexes have catalytic activity in this transformations, while others remained ineffective. A reasonable rhodium catalytic cycle involved the C–H activation of substrate 1 with the Rh(iii) catalyst to form a six-membered rhodacycle A*via* a CMD process. Next, the migratory insertion of 5 to Rh(iii) complex A afforded Rh(iii) species B, which under a β-carbon elimination gave rhodacycle C. Intramolecular nucleophilic attack of the N-atom to Rh(iii) and subsequent protonation rendered a Rh(iii) complex D, followed by reductive elimination to yield 1,2,3-trisubstituted indole 31. The generated Rh(i) was then oxidized to the catalytically active Rh(iii) species (Scheme 22). The synthetic application of this method was showed by the introduction of a carbonyl methylene group at the C7-position of indole and also the reduction of ketone to the alcohol moiety.

**Scheme 23:**
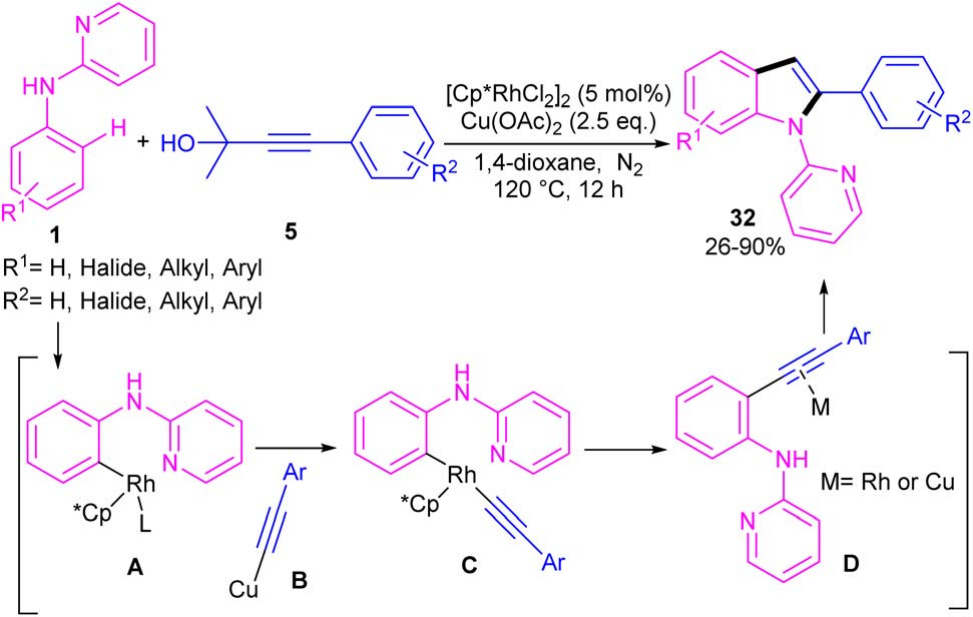
Rh(iii)-catalyzed annulation of *N*-phenyl 2-aminopyridine and propargylic alcohols.

**Scheme 24:**
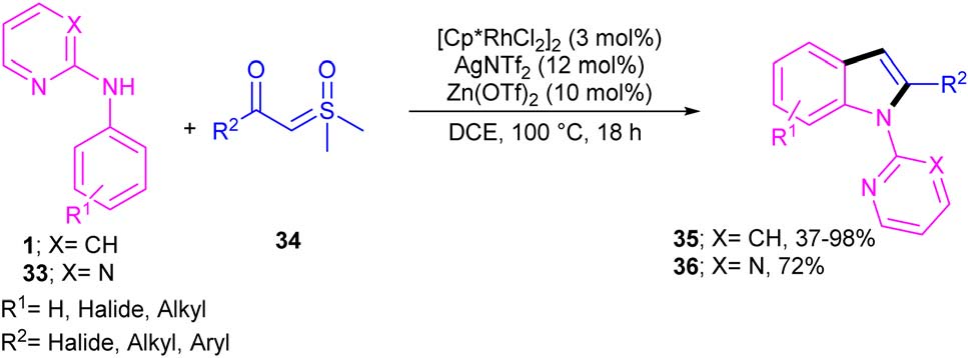
Rh(iii)-catalyzed annulation of *N*-phenyl 2-aminopyridine and sulfoxonium ylides.

**Scheme 25:**
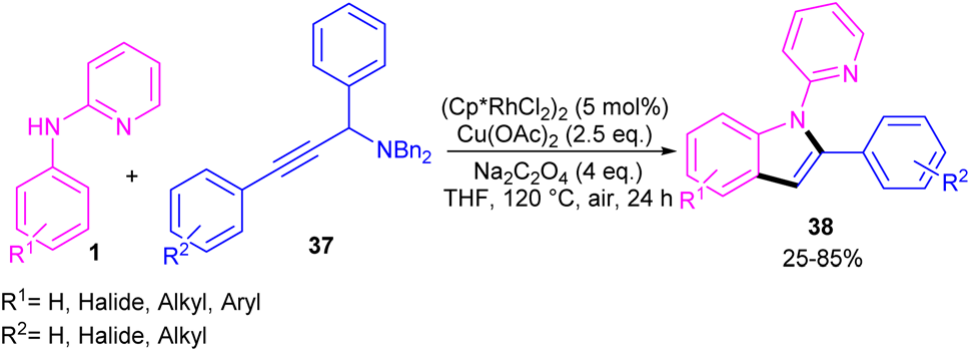
Rh(iii)-catalyzed annulation of *N*-phenyl 2-aminopyridine and propargylic amines.

Propargylic alcohols can be a practical coupling partner in the reaction with *N*-aryl-2-aminopyridines to assemble indole derivatives (Scheme 23).^41^ 5 mol% of Rh(iii) and 2.5 equiv. of Cu(ii) constitute an efficient catalytic system for the C–H activation and cascade annulation reaction. In this regard, *ortho* C(sp^2^)–H cleavage of 1 gave ruthenacycle A, which was then coordinated with the alkynyl copper species B to form an alkynyl rhodium C through transmetalation. Next, reductive elimination and subsequent annulation *via* nucleophilic attack of the amino group to the activated alkynyl moiety occurred in the presence of a Lewis acid (Rh or Cu salt) to deliver product 32. The generated Rh(i) was oxidized by Cu(ii) to the active Rh(iii) species to fulfill the catalytic cycle. None of metal catalysts including Ni(OAc)_2_·4H_2_O, Co(OAc)_2_·4H_2_O, [RuCl_2_(*p*-cymene)]_2_, and Pd(OAc)_2_ were workable. The same rhodium catalyst can also catalyze the annulation of *N*-aryl-2-aminopyridines 1 and *N*-aryl-2-aminopyrimidine 33 with sulfoxonium ylides 34 to provide indoles 35, 36 in up to excellent yields (Scheme 24).^42^ The indole synthesis involved C–H activation, coordination/insertion, and reductive elimination. The DMSO and H_2_O molecules were the only byproducts of this reaction. A similar rhodium catalytic system was reported for the reaction of *N*-aryl-2-aminopyridines 1 with propargylic amines 37 to assemble the indole products 38 (Scheme 25).^43^ A reaction mechanism similar to propargylic alcohols was suggested for this transformation, involving Rh-catalyzed C–H activation, alkyne insertion, and Lewis acid-promoted nucleophilic attack of the amino group to the activated alkynyl moiety towards intramolecular cyclization.

**Scheme 26:**
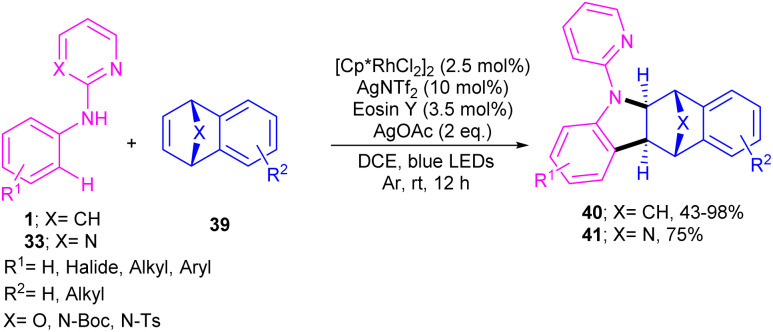
Rh(iii)-catalyzed C–H annulation of aromatic amines with bicyclic alkenes.

The cooperation of a rhodium catalysis and a photocatalysis can provide an efficient system for synthesizing a new library of bridged tetrahydro benzocarbazole scaffolds 40, 41 (Scheme 26).^44^ Various oxa- and aza-bicyclic alkenes were tolerated smoothly in the (2 + 3)-annulation reaction with *N*-aryl 2-aminopyridines 1 leading to bridged oxa-, aza-tetrahydro benzocarbazoles. The structure of bicyclic motif was intact in this reaction. Further aromatization under acidic conditions can provide the corresponding benzo[*b*]carbazoles *via* a ring-opening/hydration step. The method has the advantages of low catalyst loading, mild reaction conditions, broad functional groups tolerance and gram-scale synthesis. In addition, the removal of the directing group pyridine, acylation and Ullmann C–N coupling under copper catalysis were also carried out in this work.

**Scheme 27:**
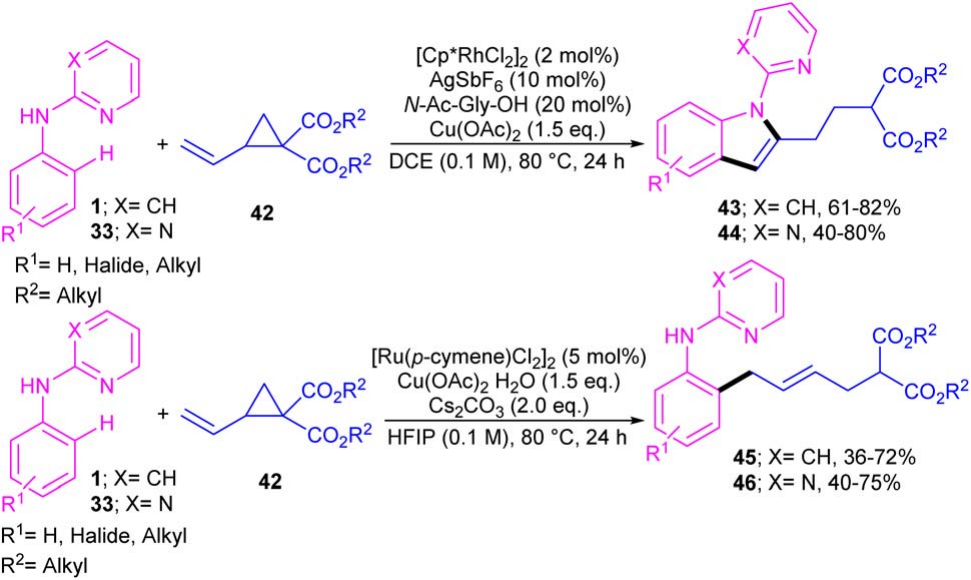
Catalyst-controlled chemodivergent approach to access C2-substituted indoles and aniline derivatives.

A series of *N*-phenyl 2-aminopyridines 1 and *N*-phenyl 2-aminopyrimidines 33 were investigated in the reaction with electron-deficient cyclopropanes 42 under metal catalysis systems (Scheme 27).^45^ Interestingly, when *N*-phenyl 2-aminopyridine used as a substrate, open-chain aniline products were obtained under ruthenium catalytic conditions. While these substrates did not reactive in the presence of a rhodium catalyst. Unlike, *N*-phenyl 2-aminopyrimidines were well tolerated in not only ruthenium-catalyzed reaction with cyclopropanes towards aniline products but also in rhodium-catalyzed annulation reaction with cyclopropanes towards indole frameworks. However, the replacement of NH-phenyl moiety with O-phenyl or S-phenyl group in the pyridine and pyrimidine substrates did not yield any products, indicating the important role of the nitrogen as a chelating group for the formation the metal complexes.

**Scheme 28:**
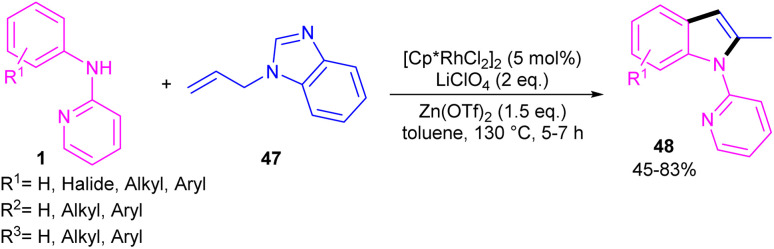
Rh(iii)-catalyzed (3 + 2)-annulation of *N*-phenyl 2-aminopyridine and *N*-allylbenzimidazole.

**Scheme 29:**
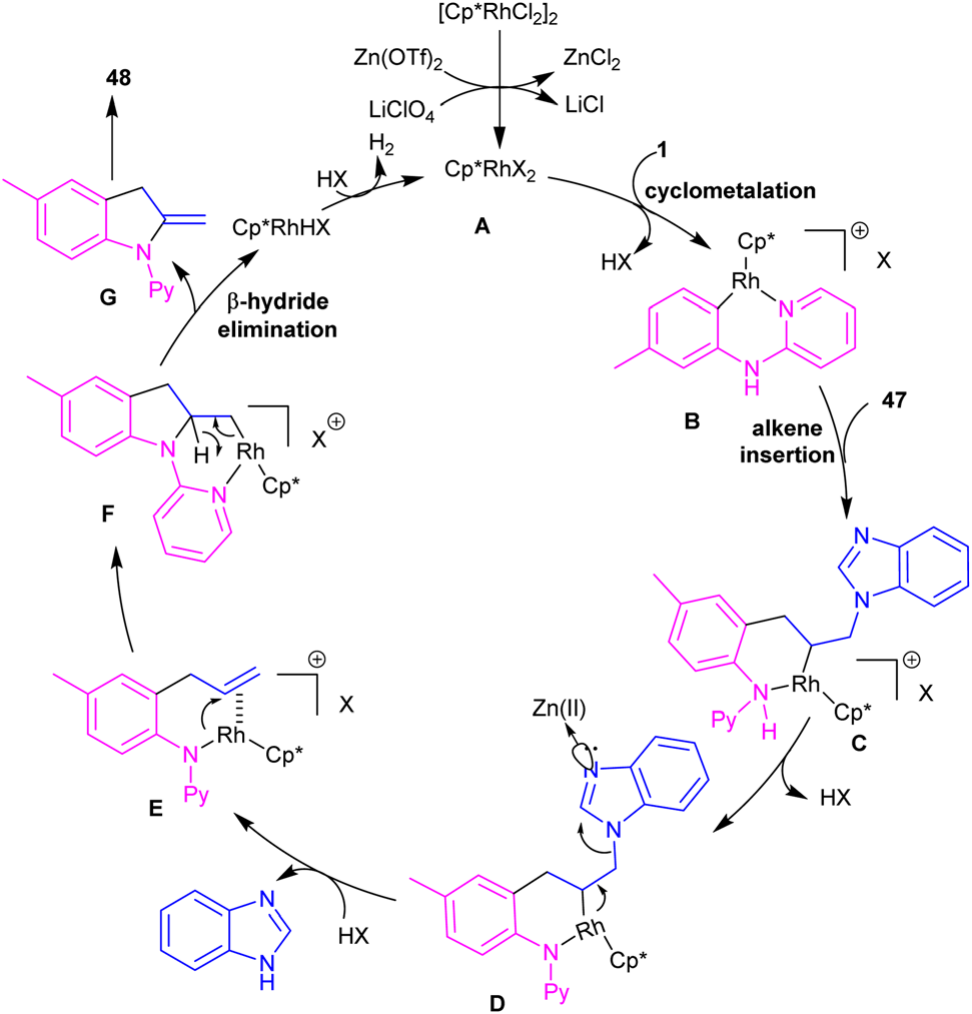
Catalytic cycle for Rh(iii)-catalyzed (3 + 2)-annulation of *N*-phenyl 2-aminopyridine and *N*-allylbenzimidazole.

2-Methylindole derivatives can be achieved from the (3 + 2)-annulation of *N*-phenyl 2-aminopyridines and *N*-allylbenzimidazoles (Scheme 28).^46^ The method involved the cleavage of C–N bond of *N*-allylbenzimidazole to act as a 2C synthon participated in the intramolecular (3 + 2)-cycloaddition with *N*-phenyl 2-aminopyridines. Radical scavenger experiments by TEMPO and BHT demonstrated a non-radical pathway. The catalytic cycle involved irreversible cyclometalation of the active catalyst A with *N*-(*p*-tolyl)pyridine-2-amine 1, followed by migratory insertion of alkene 47 into the Rh–C bond. Next, the removal of benzimidazole gave intermediate E, followed by the addition of alkene to the Rh–N bond to form intermediate F. The β-hydride elimination of F, and subsequent aromatization delivered product 48 (Scheme 29). The utility of this method was demonstrated by removal of the pyridyl group, bromination, and acetylation reactions of indole as well as the gram-scale synthesis.

#### 2.2.2. Functionalization

##### 2.2.2.1. Alkylation

**Scheme 30:**
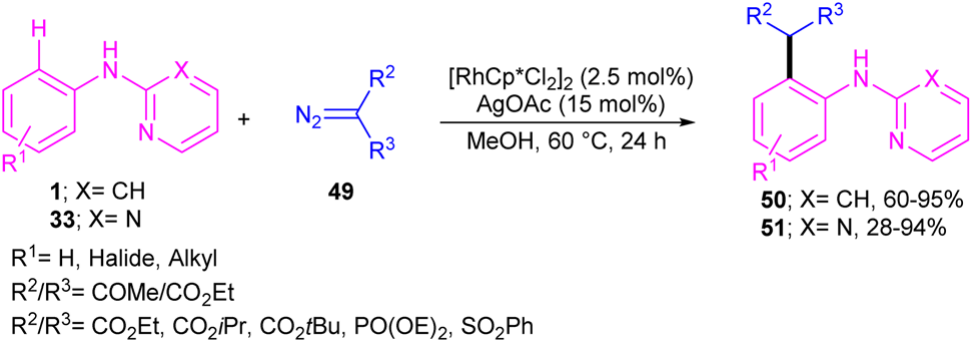
Rh(iii)-catalyzed alkylation of *N*-phenyl 2-aminopyridine with diazo compounds.

**Scheme 31:**
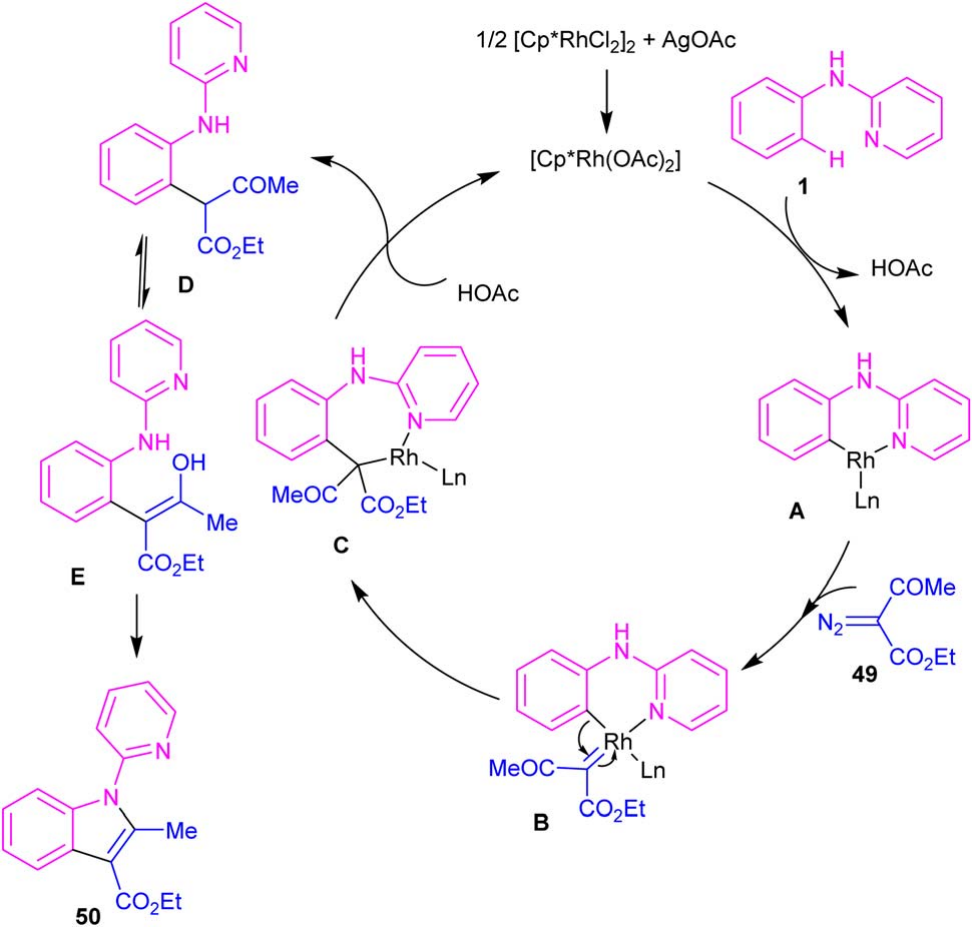
Plausible catalytic pathway for Rh(iii)-catalyzed alkylation of *N*-phenyl 2-aminopyridine with diazo compounds.

Kim and co-workers reported the cross-coupling of *N*-aryl-2-aminopyridines and *N*-aryl-2-aminopyrimidines with diazo compounds in moderate to excellent chemical yields in the presence of [RhCp*Cl_2_]_2_ (2.5 mol%) and AgOAc (15 mol%) in methanol (Scheme 30).^47^ Diazo malonates as coupling partners gave the alkylation products, while the indole products were isolated using diazo acetoacetates. In every case of alkylation, only monoalkylation product was observed, and it occurred exclusively in the C2-position. Late-stage functionalizations of the indole compounds at the C7-position, including alkylation, cyanation and amidation were also performed in this work. The mechanism of the reaction involved the generation of a six-membered rhodacycle A from 1 and the Rh(iii), followed by the coordination of α-diazo compound 49 along with release of N_2_ to form the metal-carbenoid intermediate B. Migratory insertion in B rendered the seven-membered rhodacycle C, which was protonated to generate the *ortho*-alkylated product D and the reactive Rh(iii) species. On the other hand, through a keto–enol tautomerization of D, intermediate E was obtained, which was then subjected to dehydration to afford indole 50 (Scheme 31).

##### 2.2.2.2. Thiolation

**Scheme 32:**
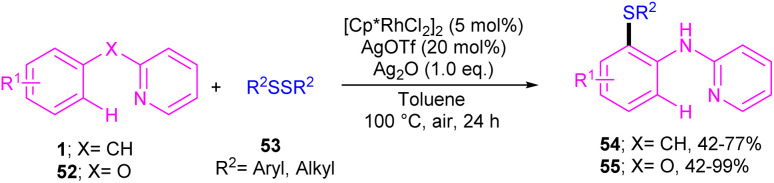
Rh(iii)-catalyzed annulation of *N*-aryl-2-pyridinamines with disulfides.

**Scheme 33:**
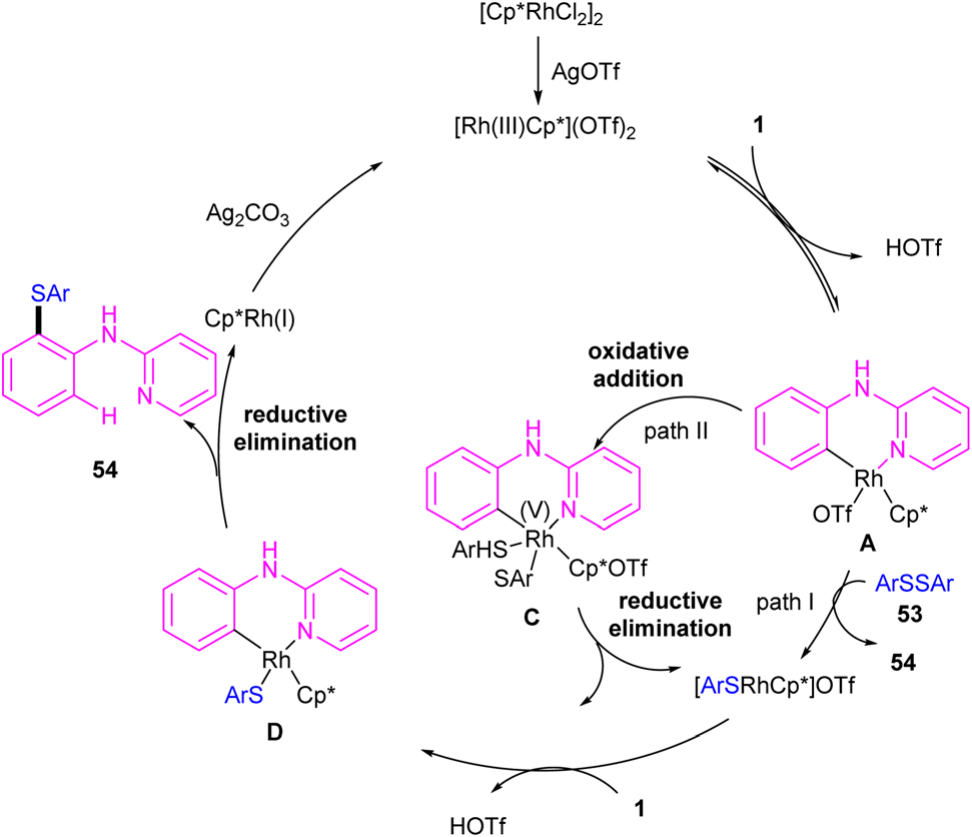
Credible mechanism for Rh(iii)-catalyzed annulation of *N*-aryl-2-pyridinamines with disulfides.

In 2017, Yang and his team realized that [Cp*RhCl_2_]_2_, AgOTf and Ag_2_CO_3_ in toluene at 130 °C can constitute an efficient catalytic system for the coupling of *N*-aryl-2-pyridinamines 1 or 2-phenoxypyridines 52 with aryl/alkyl disulfides 53 (Scheme 32).^48^ This work presented a method for the selective monothiolation of *N*-aryl-2-pyridinamines and 2-phenoxypyridines. Several mechanistic reactions were carried out to reveal the real mechanism; in a competitive reaction, electron-donating groups on the benzene ring of *N*-aryl-2-pyridinamines showed higher reactivity. Deuterium labeling experiments also confirmed the reversibility of C–H activation and the involvement of it in rate-determining step. Therefore, a six-membered rhodacyclic intermediate A was proposed to generate, which could be transformed to the rhodium(iii) intermediate C through the oxidative addition of disulfide 53. The final product 54 was obtained *via* either direct reductive elimination of C, or from the reductive elimination of the rhodacyclic intermediate D. It should be noted that intermediate D could be obtained *via* the second C–H activation 1 and B (Scheme 33).

**Scheme 34:**
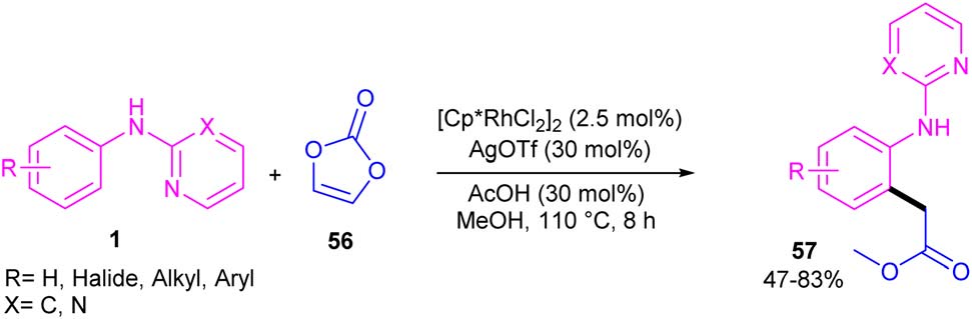
Rh(iii)-catalyzed C–H carboxymethylation of *N*-aryl-2-aminopyridines with vinylene carbonate.

Regioselective C–H carboxymethylation of *N*-aryl 2-aminopyridines 1 with vinylene carbonate 56 can be carried out in the presence of a rhodium catalyst (Scheme 34).^49^ The transformation was not successful using other metal catalysts, such as ruthenium and iridium complexes. The catalytic procedure involved C–H activation, the vinylene carbonate insertion, and 1,2-Rh–C migration/decarboxylation. The easy removal of pyridine directing group was also possible under basic conditions. In addition, the large-scale synthesis the product (1.02 g, 70% yield) and the hydrolysis of the ester to acid group showed the utility of this method.

##### 2.2.2.3. Alkenylation

**Scheme 35:**
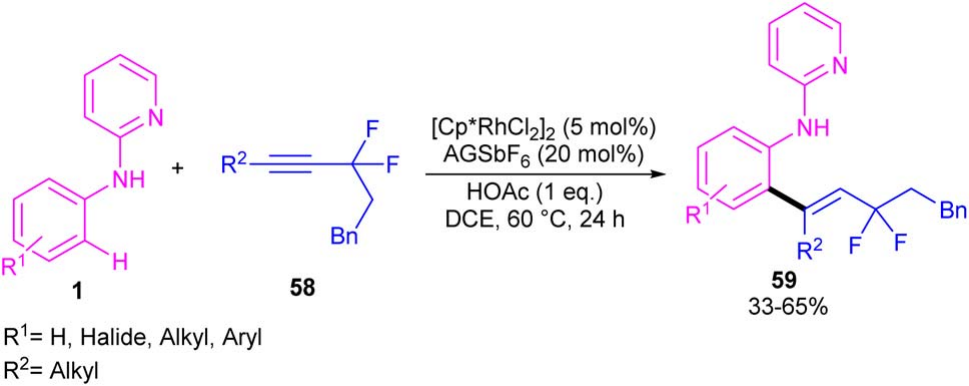
Rh(iii)-catalyzed C–H alkenylation of *N*-pyridylanilines and *gem*-difluoromethylene alkynes.

**Scheme 36:**
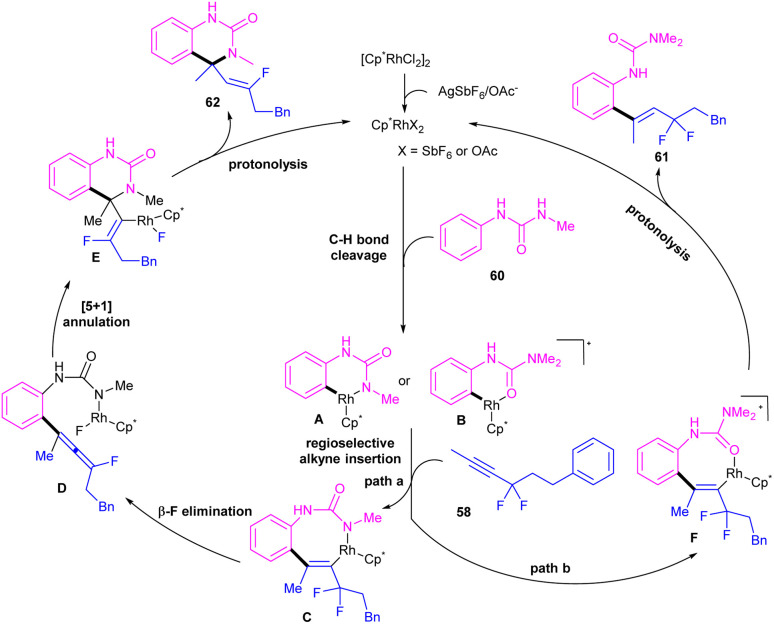
Plausible mechanism for Rh(iii)-catalyzed C–H alkenylation with *N*-pyridylanilines and *gem*-difluoromethylene alkynes.

A remarkable rhodium catalyzed C–H alkenylation of *N*-aryl-2-aminopyridine 1 with *gem*-difluoromethylene alkynes 58 was explored by Yi *et al.* (Scheme 35).^50^ Fluorine functional groups can affect the coordination mode of the Rh(iii) catalyst binding to the directing group, leading to difluorinated 2-alkenyl arylureas and 3,4-dihydroquinazolin-2(1*H*)-ones with an α-quaternary carbon center and a monofluoroalkenyl motif. Deuterium-labeling and KIE experiments indicated the reversibility of the C–H activation and non-involvement of the C–H activation in the rate-determining step. The authors assumed arylurea 60 as a substrate to design a plausible mechanism. As shown in Scheme 36, the active cationic Cp*Rh(iii) catalyst coordinated with the arylurea substrate 60 followed by the *ortho* C–H bond activation to generate the six-membered rhodacycle A or B. In path I, intermediate A moved through a regioselective migratory insertion with alkyne 58, followed by β-F elimination to form a fluoroallene intermediate D. Intramolecular (5 + 1)-cycloaddition in D gave the 3,4-dihydroquinazolin-2(1*H*)-one skeleton E, which under protonolysis delivered product 61 and the active Rh(iii) species. In path II, rhodacycle B underwent an alkyne insertion to afford intermediate F, which by further protonolysis furnished product 62.

##### 2.2.2.4. Phosphorylation

**Scheme 37:**
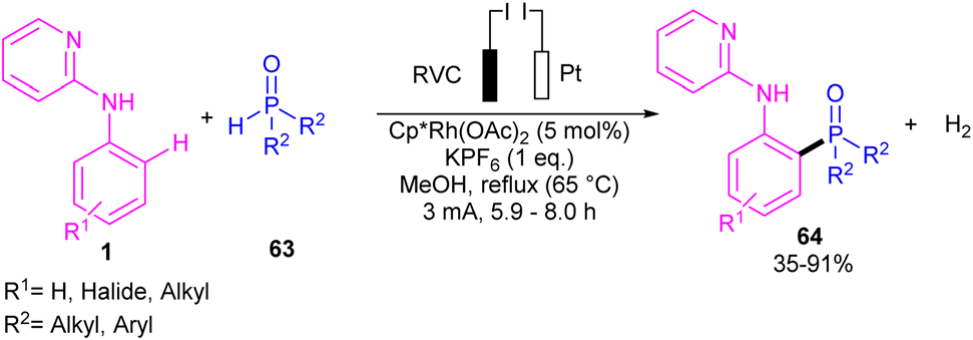
Rh(iii)-catalyzed C–H phosphorylation of *N*-aryl-2-aminopyridines with phosphorous reagents.

**Scheme 38:**
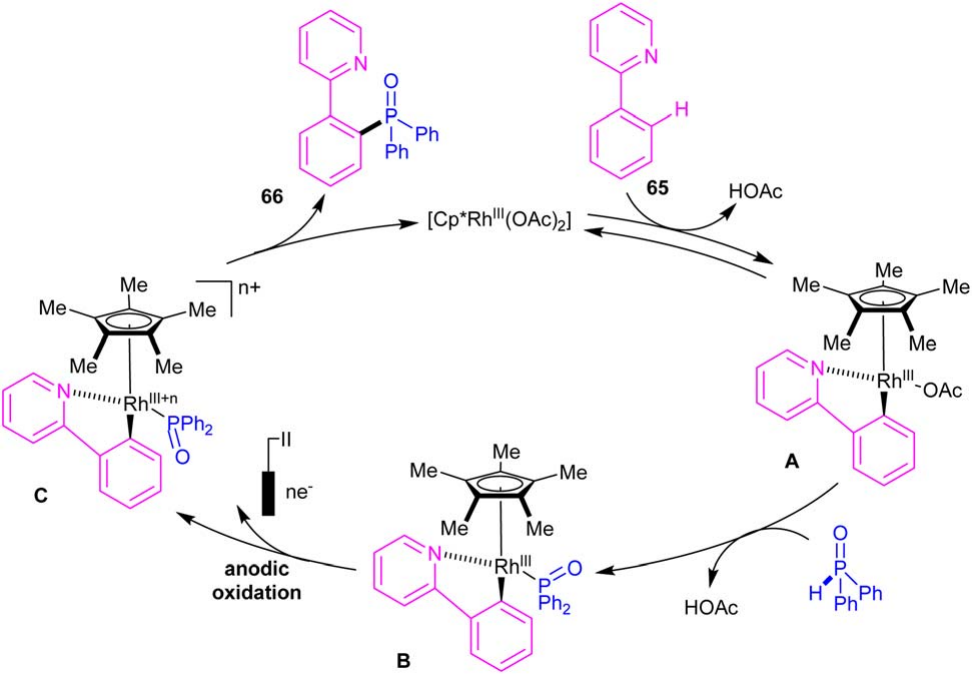
Electrocatalytic cycle for Rh(iii)-catalyzed C–H phosphorylation of *N*-aryl-2-aminopyridines with phosphorous reagents.

Phosphorylation of *N*-aryl-2-aminopyridines with a wide range of phosphorous reagents was reported under electrochemical conditions (Scheme 37).^51^ This method presented a silver-free Rh-catalyzed coupling reaction between C–H and P–H bonds. A series of triaryl/alkylphosphine oxides 64 were obtained in moderate to excellent yields. In addition to *N*-aryl-2-aminopyridine, various aryl substrates such as 2-phenylpyridine, 1-phenylpyrazole and 1-methyl-5-phenyl-1,3-dihydro-2*H*-benzo[*e*][1,4]diazepin-2-one were also incorporated in the reaction with *H*-phosphonates. The authors designed a rational mechanism using 2-phenylpyridine 65 and diphenylphosphine oxide 63 as coupling partners. The reaction was initiated by the activation of C(sp^2^)–H bond of *N*-aryl-2-aminopyridines by the metal to form rhodacycle A, which exchanged a ligand with 65 to yield a more oxidizable organometallic complex B. Next, reductive elimination of B by anodic oxidation afforded product 66 and either Rh(iii) or Rh(ii) species depending on the oxidation state of C. H_2_ molecular was produced by the reduction of protons at the cathode (Scheme 38).

### 2.3. Ir-catalyzed cross-coupling of *N*-aryl-2-aminopyridines

#### 2.3.1. Annulation

**Scheme 39:**
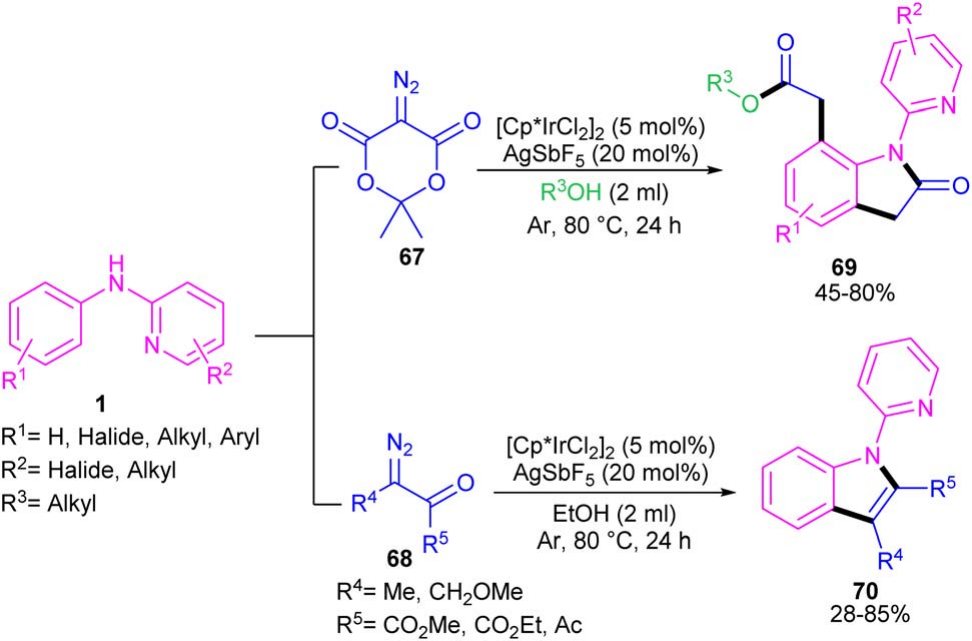
Ir(iii)-catalyzed annulation of *N*-aryl-2-pyridinamines with diazo Meldrum's acid.

**Scheme 40:**
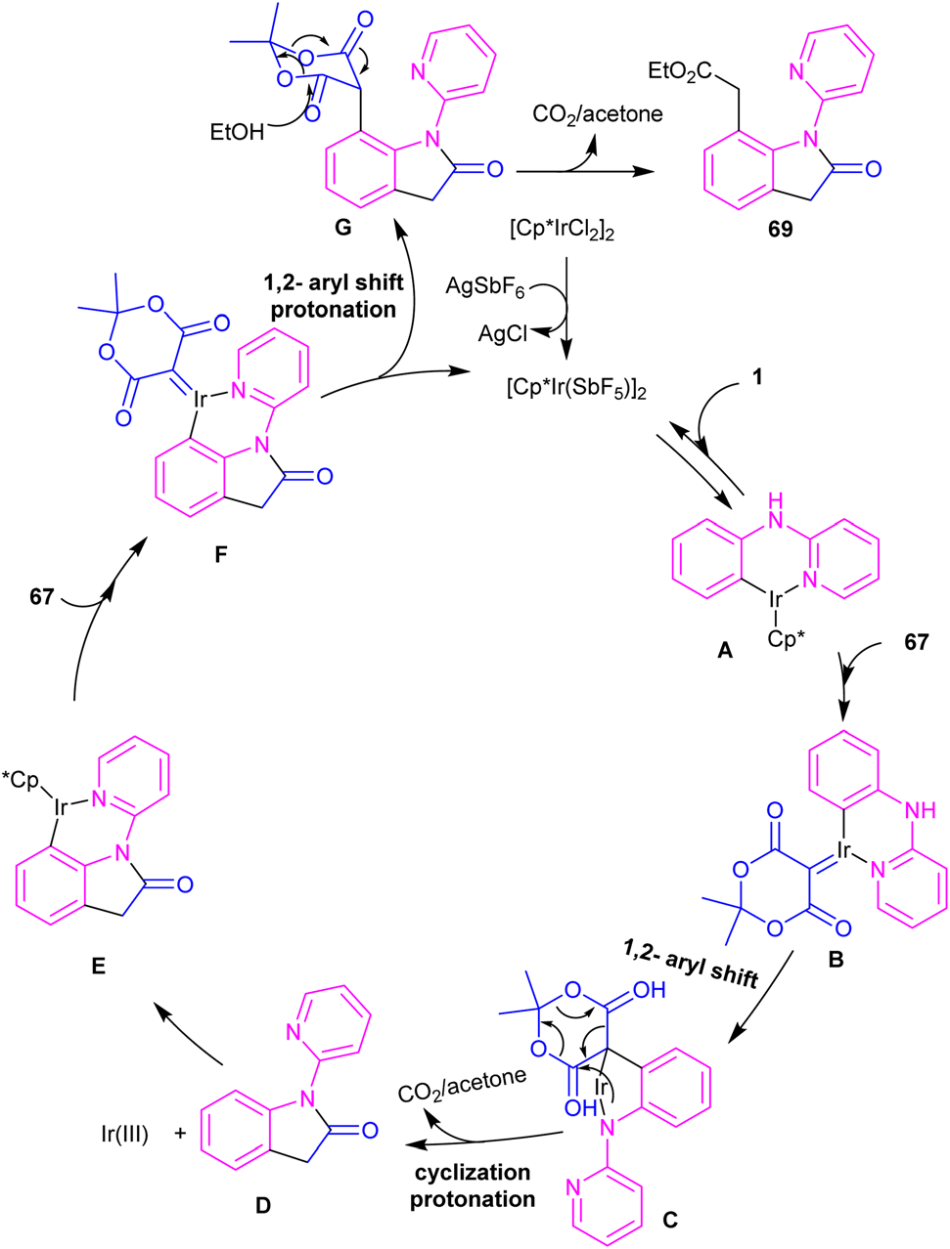
Possible mechanism for Ir(iii)-catalyzed annulation of *N*-aryl-2-pyridinamines with diazo Meldrum's acid.

**Scheme 41:**
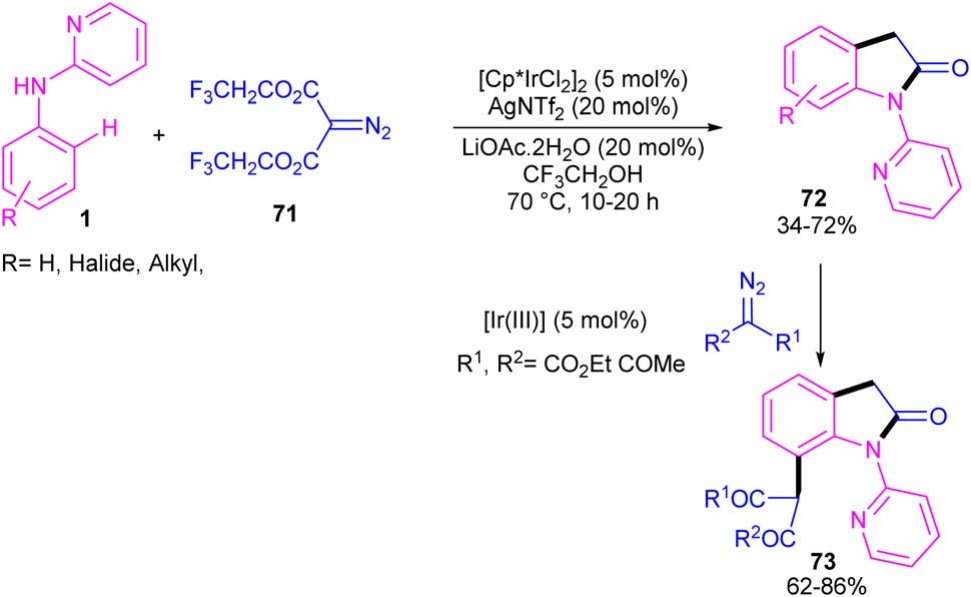
Ir(iii)-catalyzed annulation of *N*-phenyl-2-aminopyridine with bis(2,2,2-trifluoroethyl) 2-diazomalonate.

Zeng and co-workers established an iridium(iii) catalysis system for the annulation/functionalization of *N*-aryl-2-pyridinamines with diazo Meldrum's acid (Scheme 39).^52^ Various diazo compounds were investigated in the reaction with *N*-aryl-2-pyridinamines, in which 1,3-diketo-2-diazo compounds, and 1,3-ketoester-2-diazo compounds led to the corresponding indoles *via* mono selective *ortho* C–H activation. However, 1,3-diester-2-diazo compound and ethyl diazoacetate did not give any product. A possible mechanism was proposed for this carbenoid insertion/cyclization reaction, involving *N*-pyridyl coordination of 1 to the Ir(iii), and subsequent phenyl C–H activation to form a six-membered Ir(iii) complex A*via* a CMD process. Next, diazo compound 67 coordinated to the metal center of A, followed by denitrogenation to yield Ir–carbene B. After that, B underwent 1,2-aryl migratory insertion and cyclization/deprotonation to form *N*-(2-pyridyl)-2-indolone D. Finally, pyridyl-assisted the C(sp^2^)–H bond carbenoid insertion to obtain intermediate G, which nucleophilically attacked by an alcohol to provide the 7-substituted-2-oxindoles 69 with the release of acetone and CO_2_ (Scheme 40). Another work on the synthesis of oxindoles starting from *N*-phenyl-2-aminopyridine 1 with bis(2,2,2-trifluoroethyl) 2-diazomalonate 71 was described in the same year (Scheme 41).^53^ In this method, the same iridium(iii) catalyst used by Zeng, could catalyze the assembly of oxindoles and 7-substituted-2-oxindoles. The general mechanism involved pyridyl-directed C–H activation, interaction of 71 with the Ir center to form iridium–carbene bond, migratory insertion, reductive elimination and the removal of CF_3_CH_2_H and CO_2_.

**Scheme 42:**
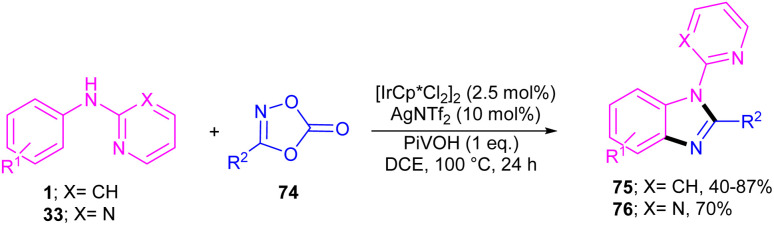
Ir(iii)-catalyzed C–H activation and amidation of aniline derivatives.

**Scheme 43:**
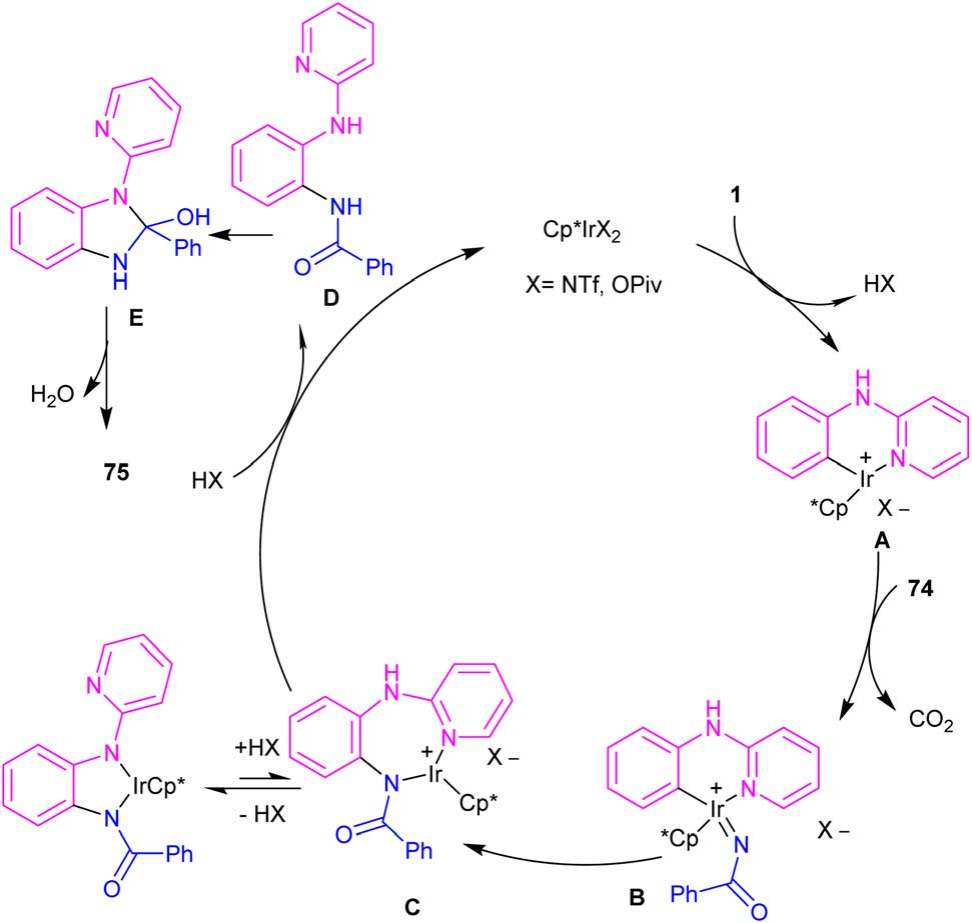
Rational mechanism for Ir(iii)-catalyzed C–H activation and amidation of aniline derivatives.

The first C–H activation and intermolecular coupling reaction access to benzimidazoles was reported by the Li group in 2017 (Scheme 42).^54^ The authors highlighted the importance of both [Cp*IrCl_2_]_2_ and a silver additive for this reaction. The reaction of *N*-aryl-2-pyridinamines and dioxazolones involved a redox-neutral mechanism and CO_2_ and H_2_O were the only byproducts. First, nitrogen-assisted C–H activation of 1 with the metal produced complex A, which further coordinated with 74 to yield a nitrenoid species B with the emission of CO_2_. Migratory insertion of nitrenoid into the Ir–aryl bond led to amidate C, which was protonated into an amidated intermediate D, together with regeneration of the Cp*Ir(iii). Eventually, PivOH or Cp*Ir(iii)-mediated intramolecular nucleophilic attack at the acyl group, followed by dehydration to furnish product 75 (Scheme 43).

**Scheme 44:**
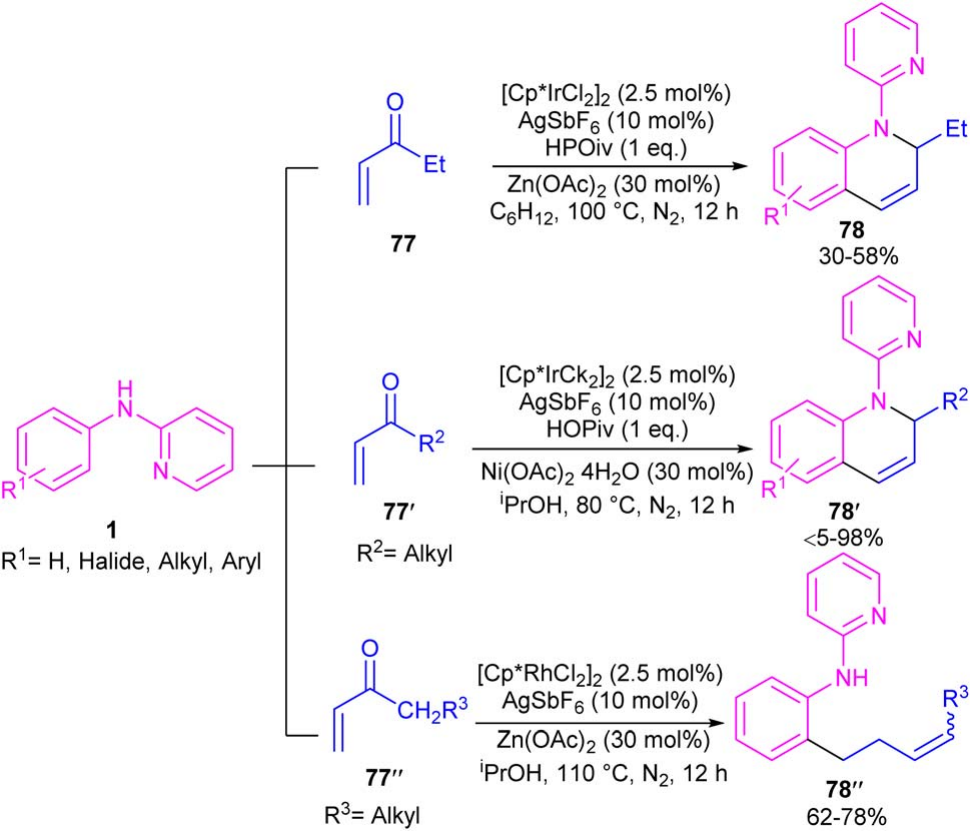
Ir(iii) and Rh(iii)-catalyzed annulation and alkylation of *N*-aryl 2-aminopyridines with enones, respectively.

**Scheme 45:**
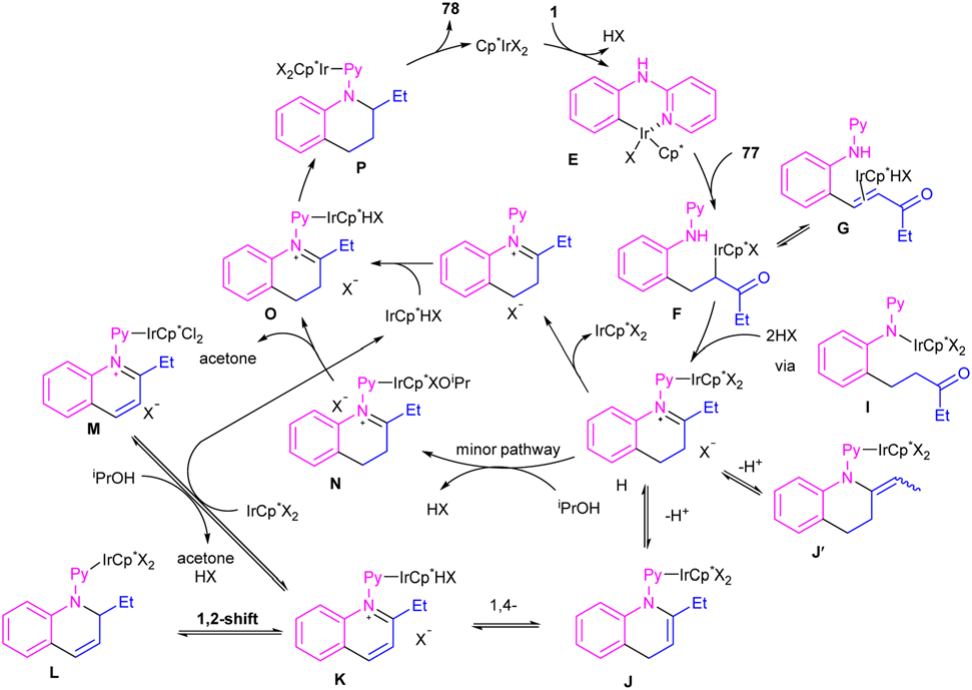
Plausible mechanism for Ir(iii)-catalyzed annulation of *N*-aryl 2-aminopyridines with enones.

By controlling of the type of the transition metal catalyst, Li co-workers were able to isolate two different products from the reaction of *N*-aryl 2-aminopyridines with enones (Scheme 44).^55^ In their work, iridium-catalyzed reaction of *N*-aryl-2-aminopyridines and enones can produce tetra- or di-hydroquinoline products, while a rhodium catalysis system including these two starting materials led to the alkylation products. It should be noted that using different iridium catalytic conditions could also lead to tetrahydroquinolines or 1,2-dihydroquinolines. It was shown that iridium catalyze the reaction through intermediate A, which under acidic media could be converted to intermediate B. Subsequent transfer hydrogenation in B provided the cyclized products. Whereas, intermediate A directly afforded the alkylation product 78′′ under rhodium catalysis. A plausible mechanism was proposed for generating 1,2-dihydroquinolines. First, after the generation of iridacycle E*via* C–H activation of aniline 1 by metal, coordination and insertion of enone 77 into the Ir–Ar bond occurred to obtain the Ir(iii) alkyl complex F in equilibrium with hydride G. After that, F was protonated and cyclized in the presence of Lewis acid Zn(ii) or Ni(ii). The obtained iminium ion H was then subjected to a ligand exchange with isopropoxide and subsequent β-H elimination to produce the Ir(iii) hydride O. Next hydride attack to O provided amine P, which was converted to product 78 after a ligand dissociation. However, in the major pathway, reversible deprotonation of H gave enamines J or J′. Intermediate J can be converted to K, and L*via* reversible 1,4- and 1,2-hydride insertion. Through a ligand dissociation–association in the metal center of H and K, intermediate M was obtained, which can be reduced by iPrOH into H. Finally, disproportionation of a dihydroquinoline (J/L), and subsequent reduction of the quinolinium K delivered product 78 (Scheme 45).

**Scheme 46:**
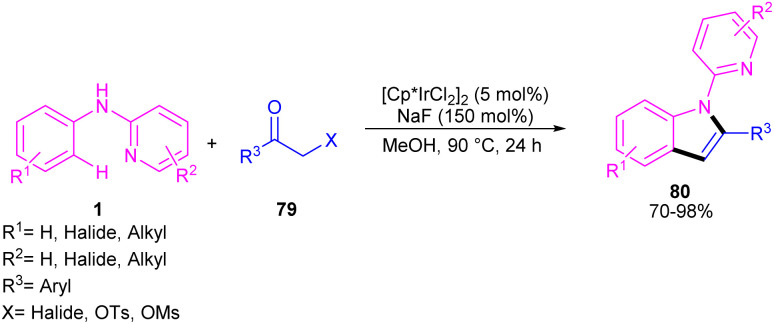
Ir(iii)-catalyzed C–H annulation of *N*-phenyl-2-aminopyridines with α-chloro ketones.

The indole synthesis can be achieved from the annulation of *N*-aryl-2-aminopyridines 1 with α-chloro ketones 79 under iridium catalysis (Scheme 46).^56^ The reaction was not proceeded in the presence of [Ru(*p*-cymene)Cl_2_]_2_ and Cp*Co(CO)I_2_, while led to 15% of chemical yield using [RhCp*Cl_2_]_2_. The transformation did not significantly influence by the electronic properties of substituents at the both benzene and pyridyl ring of *N*-aryl-2-aminopyridines, where the all products were obtained in high to excellent yields. Moreover, a wide range of α-chloro ketones with aryl, naphthyl, and heteroaryl moieties were compatible.

#### 2.3.2. Functionalization

##### 2.3.2.1. Alkenylation

**Scheme 47:**
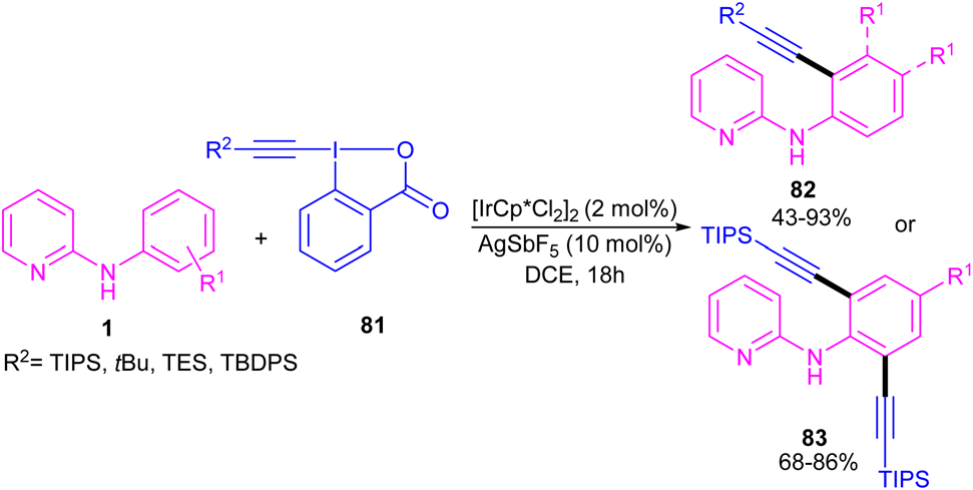
Ir(iii)-catalyzed C–H alkynylation of *N*-phenyl-2-aminopyridines.

**Scheme 48:**
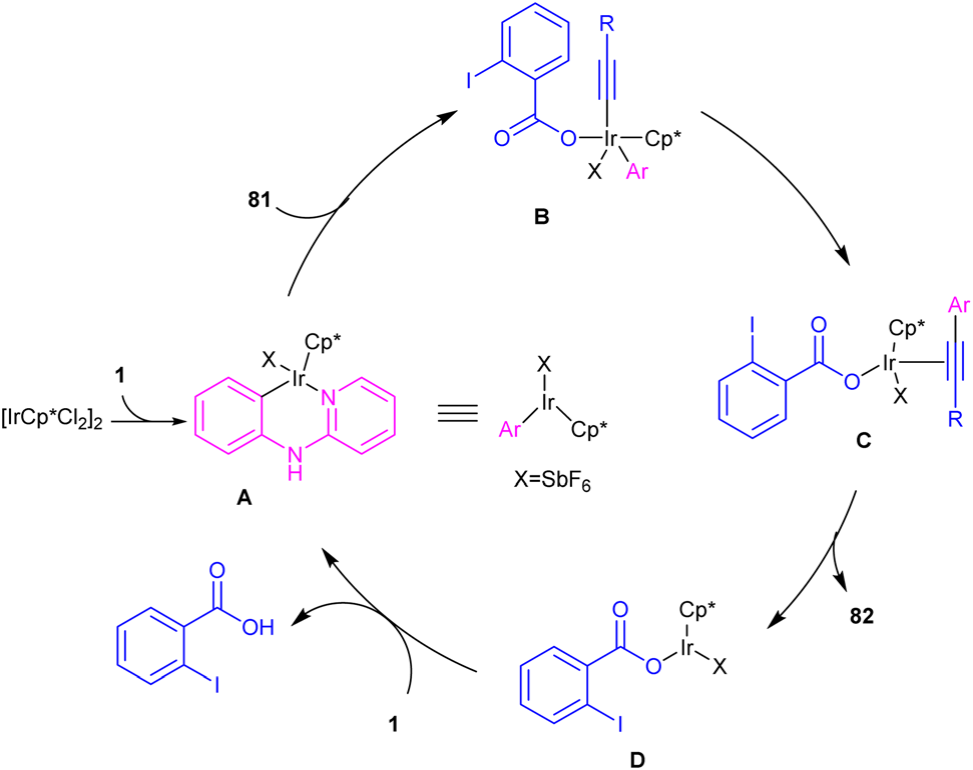
Catalytic cycle for Ir(iii)-catalyzed C–H alkynylation of *N*-phenyl-2-aminopyridines.

In 2016, *N*-phenyl-2-aminopyridines were investigated for the alkynylation in the presence of a hypervalent iodine alkyne (TIPS-EBX) as a coupling partner (Scheme 47).^57^ Iridium(iii) could efficiently catalyze the *ortho*-C–H alkynylation of *N*-phenyl-2-aminopyridines. Selectivity of mono alkynylation can be better achieved using TIPS-EBX compared to other alkyne sources. However, *ortho*- and *meta*-substituted benzene ring led to selective mono-alkynylation, while *para*-substituted benzene gave dialkynylated products. The reaction mechanism involved the formation of an active cationic Ir(iii) species from the ligand exchange between [IrCp*Cl_2_]_2_ and AgSbF_6_, followed by the coordination of metal center with 1 to produce a six-membered iridacycle A. Oxidative addition of R-EBX 81 to A gave Ir(v) intermediate B, which under reductive elimination afforded Ir(iii) alkyne C. Another C–C reductive elimination led to Rh(iii) benzoic species D and the coupling product 82. Subsequently C–H activation of 1 resulted in the active iridacycle A and 2-iodobenzoic acid (Scheme 48). KIE experiment suggested the C–H activation is the rate-determining step.

##### 2.3.2.2. Borylation

**Scheme 49:**
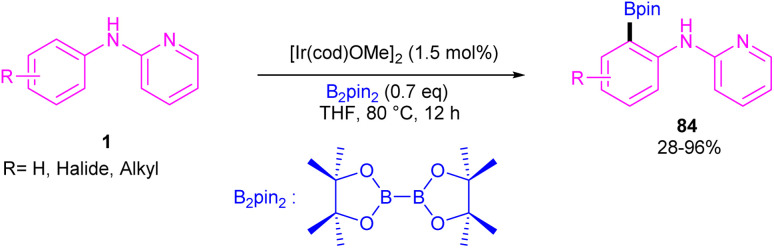
Ir(iii)-catalyzed C–H borylation of 2-anilinopyridines and 2-phenoxypyridines.

**Scheme 50:**
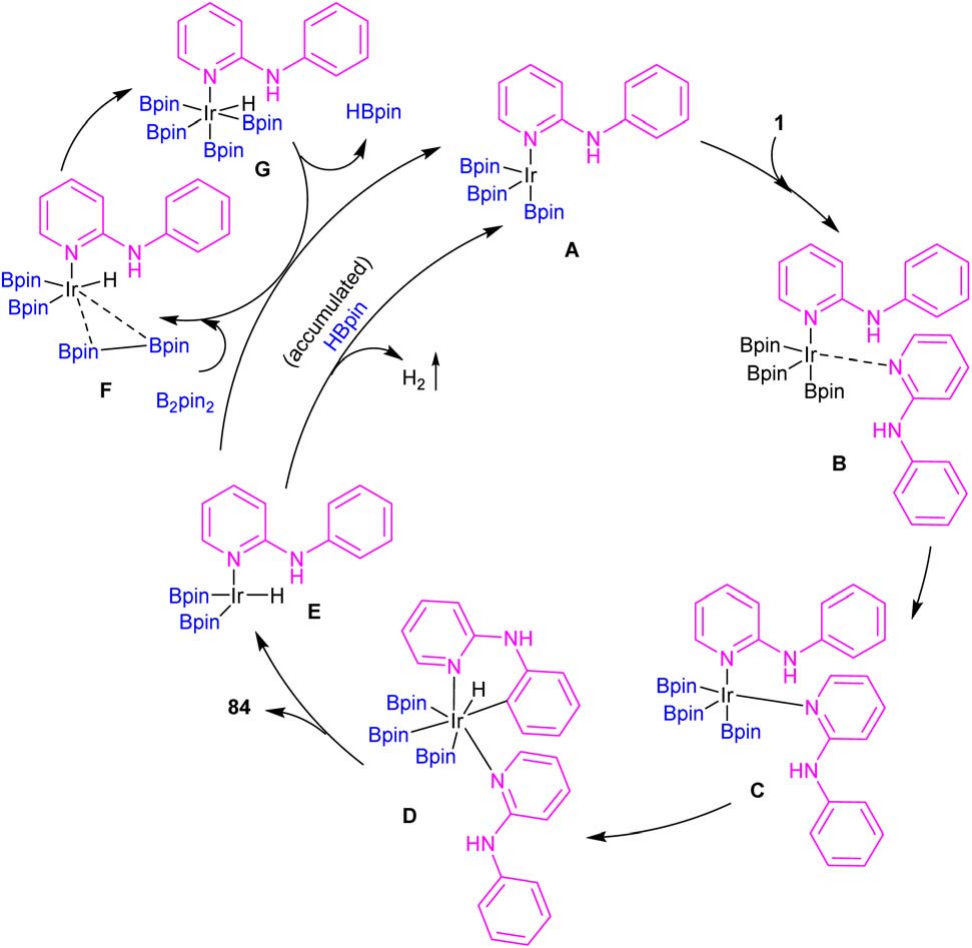
Catalytic cycle for Ir(iii)-catalyzed C–H borylation of 2-anilinopyridines and 2-phenoxypyridines.

An iridium catalysis system for the *ortho*-selective borylation of 2-anilinopyridines and 2-phenoxypyridines was developed by Chattopadhyay and his team in 2022 (Scheme 49).^58^ By decreasing the temperature, the authors were able to control the mono-borylation over di-borylation reaction. So, in lower temperatures mono-borylated products were the main products, whereas the higher temperatures were suitable for the di-borylation reaction. The borylated products were isolated up to excellent yields. Besides, other arenes, including benzylamines/piperazines/morpholines/pyrrolidine/piperidines/azepanes, α-amino acids, aminophenylethanes were participated in this catalytic system. According to KIE and DFT studies, a rational catalytic cycle was suggested involving a key iridium–boryl intermediate. Two molecules of substrate 1 coordinated with iridium to give intermediate A, which led to the iridium complex Ir(1)_2_(Bpin)_3_C. Then, oxidative addition of Ir(iii) into the *ortho* C–H bond provided Ir(v)–aryl intermediate D. Intermediate D then under reductive elimination delivered the borylated product 84 and the Ir(iii)–H intermediate E. The regeneration of the active catalyst involved the attachment of B_2_pin_2_ to intermediate E to form complex F. B_2_pin_2_ inserted to the Ir center in F to yield the hexa-coordinate Ir(v) intermediate G. Finally, the removal of HBpin through reductive elimination resulted in the active catalyst A. This HBpin molecule can be inserted to the Ir center of intermediate E, releasing H_2_*via* reductive elimination to obtain the active catalyst A (Scheme 50).

### 2.4. Ru-catalyzed cross-coupling of *N*-aryl-2-aminopyridines

#### 2.4.1. Annulation

**Scheme 51:**
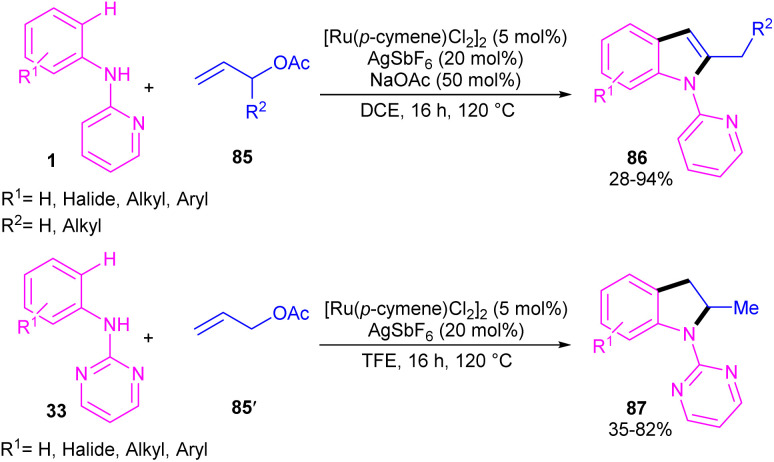
Ru(ii)-catalyzed C–H allylation/oxidative annulation of *N*-aryl-2-aminopyridines or *N*-aryl-2-aminopyrimidines with electron-deficient alkenes.

**Scheme 52:**
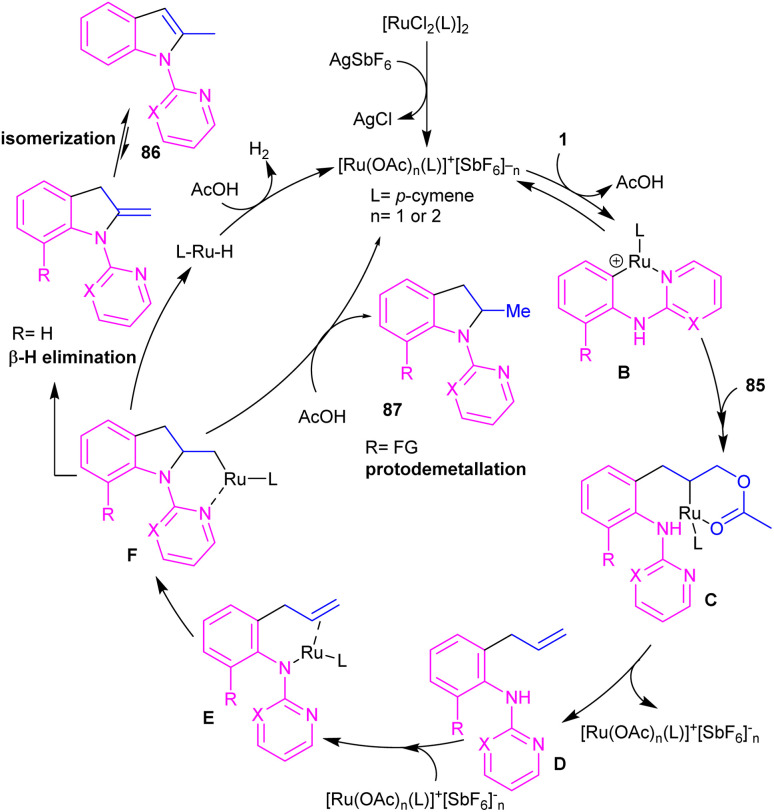
Plausible mechanism for Ru(ii)-catalyzed C–H allylation/oxidative annulation of *N*-aryl-2-aminopyridines or *N*-aryl-2-aminopyrimidines with electron-deficient alkenes.

A cascade C–H allylation/oxidative annulation of *N*-aryl-2-aminopyridines or *N*-aryl-2-aminopyrimidines with electron-deficient alkenes was reported by Jana and his team in 2018 (Scheme 51).^59^ In the case of *N*-aryl-2-aminopyridines as a substrate, β-hydride elimination from the σ-alkyl-Ru intermediate led to 2-methylindoles, while for *N*-aryl-2-aminopyrimidine substrates, due to the suppression of β-hydride elimination by steric effect in interaction with the pyrimidine group which stabilized the alkyl-Ru species through coordination, a protodemetalation occurred *in lieu* of β-hydride elimination. The authors proposed a mechanism for the Ru(ii)-catalyzed cascade reaction as shown in Scheme 52. It was proposed that *ortho* C–H activation of 1 with the active ruthenium complex generated intermediate B, which underwent migratory insertion of alkene to obtain an alkyl ruthenium intermediate C. Then, β-acetoxy elimination gave allylation intermediate D. Further coordination of the Ru with the nitrogen and subsequent alkene insertion to the allyl group resulted in intermediate F. In the case of *ortho*-unsubstituted *N*-aryl-2-aminopyrimidines, F underwent a rapid β-hydride elimination and double bond isomerization to yield product 86. Whereas, for the *ortho*-substituted *N*-aryl-2-aminopyrimidines 33, the protodemetalation in the protic solvent, like TFE, afforded product 87. The generated ruthenium hydride species in this step was converted to the active catalyst *via* the interaction of H_2_ gas with AcOH. Indole derivatives can also be achieved through ruthenium-catalyzed annulation of *N*-aryl-2-aminopyridines with α-carbonyl sulfoxonium ylides.^60^ However, [IrCp*Cl_2_]_2_ and [RhCp*Cl_2_]_2_ resulted in very low yield of product, and Cp*Co(CO)I_2_ did not workable.

#### 2.4.2. Functionalization

##### 2.4.2.1. Acylation and acetoxylation

**Scheme 53:**
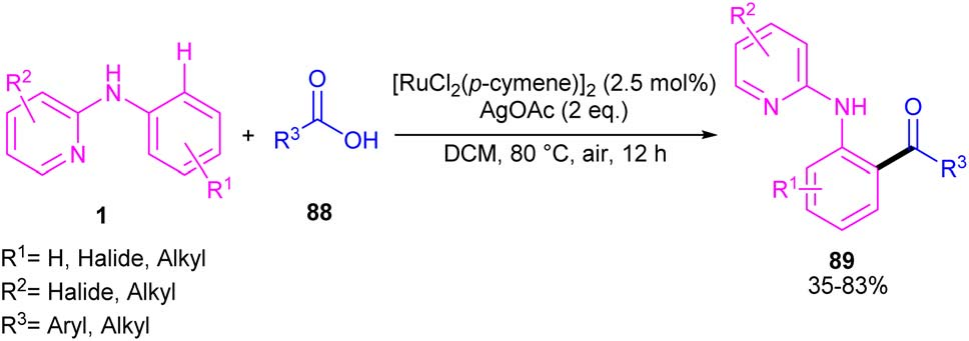
Ru(ii)-catalyzed C–H acylation of aniline derivatives with α-oxocarboxylic acids.

**Scheme 54:**
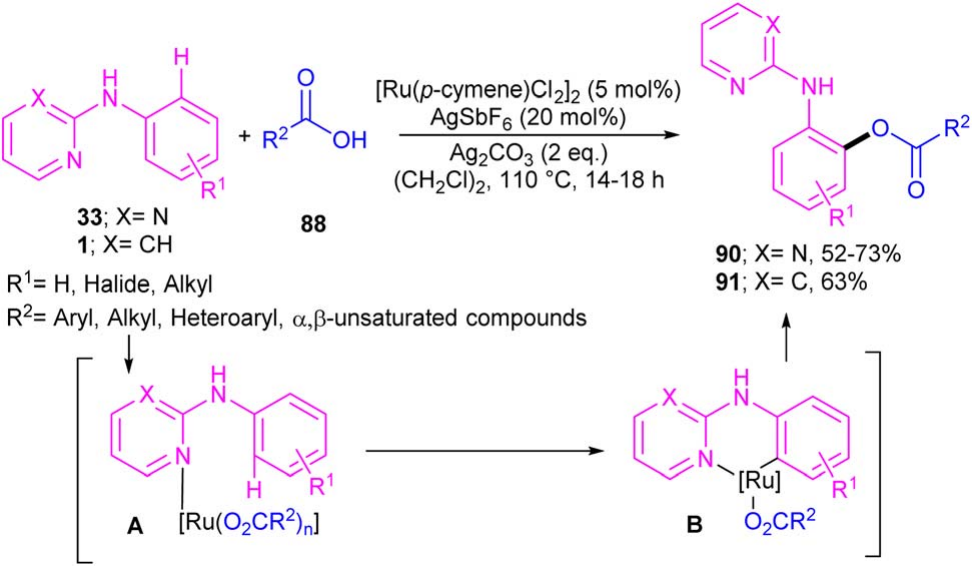
Ru(ii)-catalyzed C–H acylation of *N*-aryl-2-aminopyridines or *N*-aryl-2-aminopyrimidines with carboxylic acids.

Li and co-workers described *ortho*-acylation of *N*-(2-pyridyl)-anilines 1 with arylglyoxylic acids 88 under ruthenium catalysis (Scheme 53).^61^*N*-(2-Pyridyl)-anilines bearing electron-donating and aryl and heteroaryl glyoxylic acids electron-withdrawing groups at the both pyridine and benzene ring were evaluated, in which electron-rich substrates showed better reactivity than electron-deficient ones. In the case of arylglyoxylic acids, a certain order was not realized, while heteroarylglyoxylic acids like 2-thiopheneglyoxylic acid and 2-furanglyoxylic acid led to slightly lower products. Overall reaction mechanism involved pyridine-assisted direct C(sp^2^)–H bond activation *via* CMD pathway, acid coordination, decarboxylation, and reductive elimination. The ruthenium/silver catalysis was found to be workable for acylating *N*-aryl-2-aminopyridine or *N*-aryl-2-aminopyrimidines (Scheme 54).^62^ Carboxylic acids were selected as acylating reagents, which were coordinated with the metal center of complex A, followed by the reductive elimination towards the acylated product 90, 91.

### 2.5. Co-catalyzed cross-coupling of *N*-aryl-2-aminopyridines

#### 2.5.1. Nitration

**Scheme 55:**
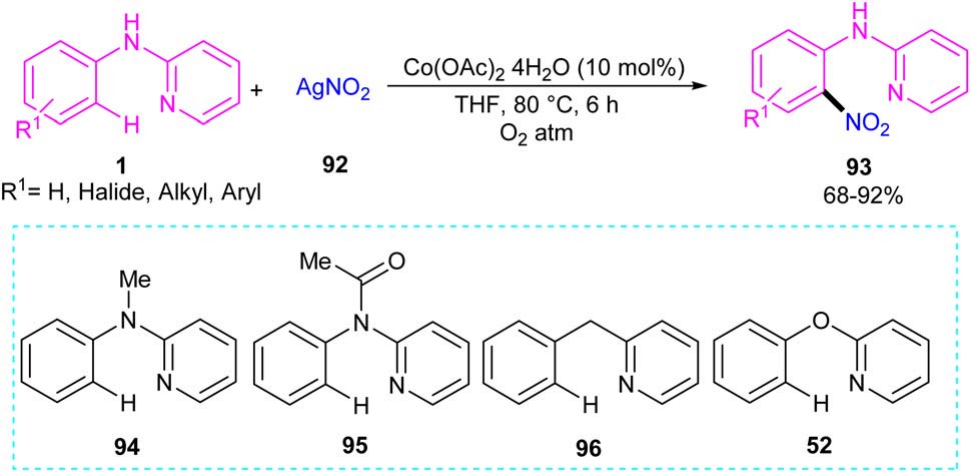
Co(ii)-catalyzed regioselective C(sp^2^)–H bond nitration of *N*-phenyl-2-aminopyridine.

**Scheme 56:**
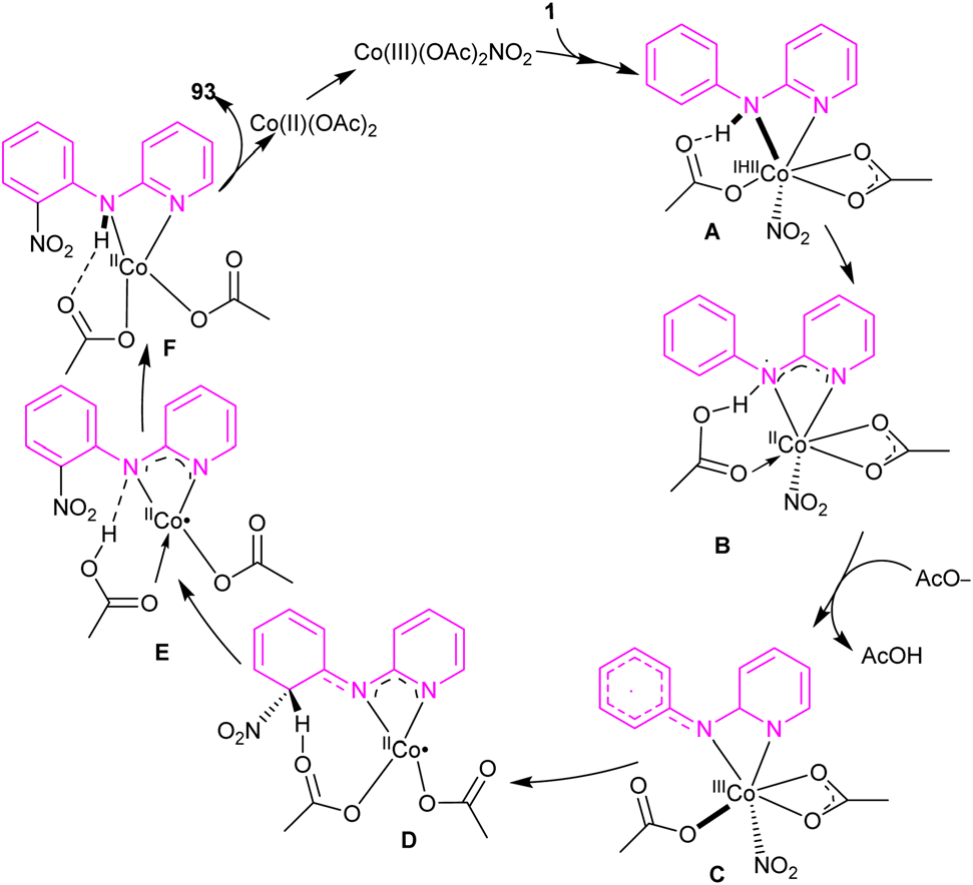
Catalytic cycle for Co(ii)-catalyzed regioselective C(sp^2^)–H bond nitration of *N*-phenyl-2-aminopyridine.

An efficient nitration method for *N*-phenyl-2-aminopyridine 1 was described by Das's group in 2017 (Scheme 55).^63^ By using an Co(OAc)_2_ catalyst, nitration of *N*-phenyl-2-aminopyridine with AgNO_3_ was achieved in good yields (70–92%). *N*-Thiophene-2-aminopyridine was also nitrated under cobalt catalysis in 68% of yield. The cobalt acetate can also catalyze the methoxylation of *N*-phenyl-2-aminopyridine using methanol as both a coupling partner and solvent. Mechanistic experiments were performed, and these allowed the authors to conclude that the reaction proceeded through a radical pathway and the C–H activation was not the rate-determining step. Interestingly, none of the substrates 94, 95, 96, and 52 were not compatible which proved the vital role of the NH in the coordination chemistry with the metal. The mechanism of the reaction started with the generation of reactant complex (RC) from the active catalyst Co(iii)(OAc)_2_(NO_2_) and *N*-aryl-2-aminopyridine. Through a PCET reaction, a proton in RC transferred to acetate, leading to the reduction of Co(iii) reduced to Co(ii). Due to the high energy barrier for the nitro group transfer from Co(ii) to phenyl group, acetic acid group exchanged with an acetate ion to form a Co(iii) complex 12. The transfer of nitro functional group from I2 gave complex 13. In I3, H-transfer reaction offered intermediate I4. Further proton transfer to the imino-functional group from AcOH provided intermediate I5. Finally, complex I5 converted to the nitrated product after the release of Co(OAc)_2_ (Scheme 56).

### 2.6. Cu-catalyzed cross-coupling of *N*-aryl-2-aminopyridines

#### 2.6.1. Annulation

**Scheme 57:**
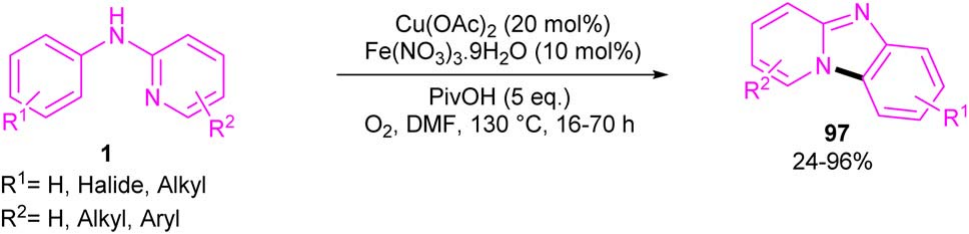
Cu(ii)-catalyzed intramolecular C–H amination of *N*-phenyl-2-aminopyridine.

**Scheme 58:**
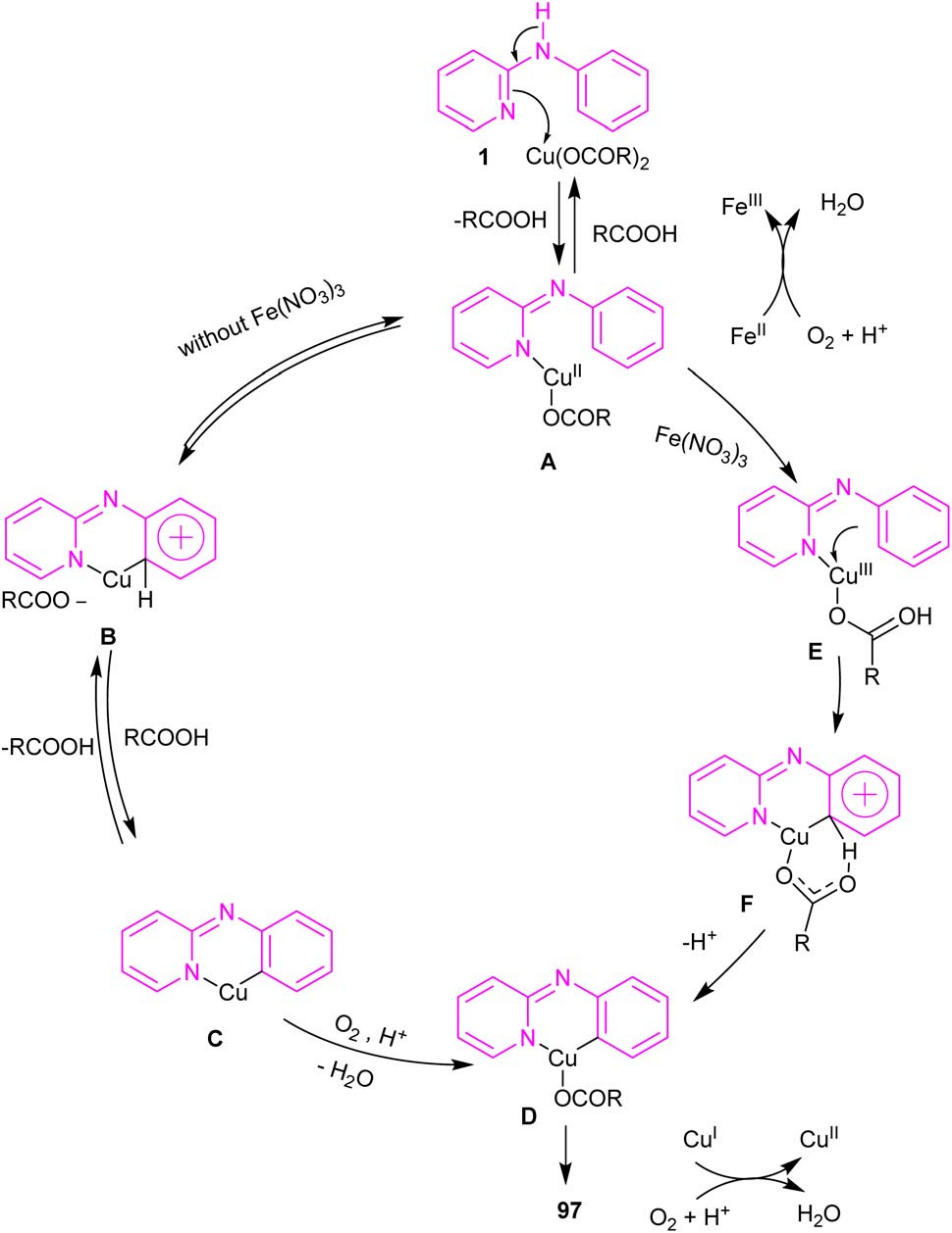
Proposed reaction pathways for Cu(ii)-catalyzed intramolecular C–H amination of *N*-phenyl-2-aminopyridine.

An intramolecular C–H amination of *N*-phenyl-2-aminopyridine derivatives was established using Cu(OAc)_2_ as a catalyst (Scheme 57).^64^ The pyridinyl nitrogen was found to be both a directing group and a nucleophile. Screening of this transformation in the presence of Fe(NO_3_)_3_·9H_2_O and without it, showed the significant role of Fe(NO_3_)_3_·9H_2_O in increasing of the reaction efficiency. The iron(iii) helped in forming the electrophilic Cu(iii) species, leading to the subsequent S_E_Ar reaction. In this context, two possible pathways were proposed in the presence and absence of iron(iii). In the first case, the Cu(ii) complex A was oxidized to the more electrophilic Cu(iii) intermediate E, followed by sequentially electrophilic substitution, and elimination of the aromatic proton to yield intermediate D. Reductive elimination then took place before protonation to furnish pyrido[1,2-*a*]benzimidazoles 97. However, without Ir(iii) salt, the Cu(ii) complex A underwent an electrophilic aromatic substitution to render the Cu(ii) intermediate C. After that, C was protonated before the oxidation process. The generated active intermediate D was then converted to product 97*via* reductive elimination. The oxidation of Cu(i) to Cu(ii) was carried out by O_2_ (Scheme 58).

#### 2.6.2. Arylation

**Scheme 59:**
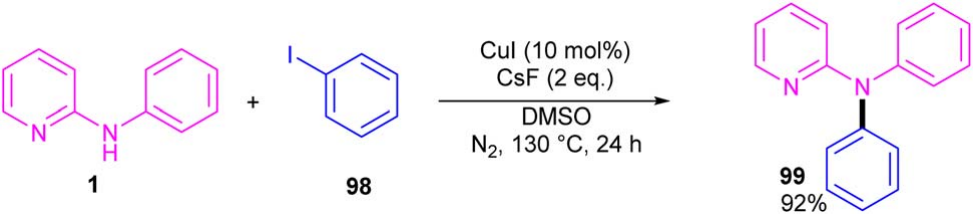
Cu(i)-catalyzed *N*-arylation of *N*-phenyl-2-aminopyridine.

Copper-catalyzed *N*-arylation of *N*-phenyl-2-aminopyridine 1 was reported by Ribas and Güell in 2014 (Scheme 59).^65^ The C–N cross-coupling was carried out in the presence of 10 mol% of CuI and 2 equiv. of CsF in DMSO. This method has presented a ligand-free Ullmann-type C–N bond coupling. DMSO served both as a solvent and ligand, overcoming the need for any external auxiliary ligands. A wide range of amines exhibited good compatibility in this methodology.

## 3. Conclusions

In this review, we presented developments on *N*-aryl-2-aminopyridines reactions under transition metal catalysis systems. As shown in this context, the nitrogen atom in the pyridyl group structure can form a series of more stable complexes with metals, leading to annulation or functionalization of *N*-aryl-2-aminopyridines. Various transition metal catalysts, such as Pd, Rh, Ir, Ru, Co and Cu salts can efficiently catalyze the transformations involving *N*-aryl-2-aminopyridines. Despite the great developments, these synthetic methods still face some disadvantages, such as high temperatures, the use of external oxidants and additives for the C–H activation reactions. Hence, designing greener and milder procedures in this field is highly desirable. In particular, the functionalization of *N*-aryl-2-aminopyridines still requires a lot of work.

Considering the sustainability and effectiveness of photocatalysis and electrocatalysis systems, it is worth mentioning that the combination of these two catalytic systems with metal containing systems can constitute an efficient multiple catalytic system for transforming *N*-aryl-2-aminopyridines.

We hope that this review will provide new insights into the chemistry and transformations of *N*-aryl-2-aminopyridines to design more practical and sustainable methods in the field of N-heterocyclic chemistry.

## Data availability

The data supporting the findings of this study are available within the article.

## Conflicts of interest

There are no conflicts to declare.
